# DESpace2: detection of differential spatial patterns in spatial omics data

**DOI:** 10.1093/bioinformatics/btag450

**Published:** 2026-08-21

**Authors:** Peiying Cai, Mark D Robinson, Simone Tiberi

**Affiliations:** Department of Molecular Life Sciences and Swiss Institute of Bioinformatics, University of Zurich, Zurich, Switzerland; Department of Molecular Life Sciences and Swiss Institute of Bioinformatics, University of Zurich, Zurich, Switzerland; Department of Statistical Sciences, University of Bologna, Bologna, Italy

## Abstract

**Motivation:**

Spatially resolved transcriptomics (SRT) enables the investigation of mRNA expression in a spatial context. While several SRT analysis frameworks have been developed, the vast majority of them focus on analyzing individual samples, and do not allow for comparisons of spatial gene expression patterns across experimental conditions, such as healthy *vs.* diseased states.

**Results:**

Here, we present an approach to identify so-called differential spatial patterns (DSP), i.e. genes that exhibit changes in spatial expression between groups of samples across conditions. Our framework processes diverse SRT data types and detects DSP by performing differential gene expression testing across conditions. Notably, this comparison is not currently available in any other spatial omics method. In addition to detecting DSP across conditions, our framework includes two key features. First, it can identify tissue regions where expression changes across conditions. Second, it can detect spatial gene expression pattern changes across more than two experimental conditions, with flexible models. With *ad hoc* simulations, we demonstrate that our approach has good true positive rates and well-calibrated false discovery rates. Applied to experimental data, our method identifies biologically relevant DSP genes while maintaining computational efficiency.

**Availability:**

Our framework has been implemented within *DESpace* Bioconductor R package (from version 2.0.0).

## 1 Introduction

Spatially resolved transcriptomics (SRT) technologies measure gene expression levels while preserving the tissue spatial context, within the local aggregate of multiple cells (termed spots), at the single-cell level, or even at the subcellular level. This enables the investigation of spatial gene expression patterns (Sun *et al.* 2020, Shang and Zhou 2022, Tian *et al.* 2023), uncovers cellular heterogeneity (Li *et al.* 2023), facilitates neighborhood analysis (Dries *et al.* 2021), enhances our understanding of disease mechanisms (Chen *et al.* 2020, Fomitcheva-Khartchenko *et al.* 2022, Zhou *et al.* 2023), and even allows for RNA expression analysis at the subcellular level (Cassella and Ephrussi 2022).

To fully leverage the potential of SRT data, several analytical methods have been developed. Among these, two standard steps are the identification of spatial domains (also known as spatial clustering), and the detection of spatially variable genes (SVGs). While single-cell RNA sequencing data allows for cell grouping based on transcriptional profiles, SRT data clustering integrates both mRNA information and spatial context to identify spatial regions of coherent gene expression within a tissue. To date, a wide range of spatially-aware clustering methods have been developed for this purpose, including *BayesSpace* (Zhao *et al.* 2021), *Banksy* (Singhal *et al.* 2024), *BASS* (Li and Zhou 2022), *STAGATE* (Dong and Zhang 2022), and *GraphST* (Long *et al.* 2023). These methods utilize diverse modeling strategies, such as graph-based deep learning (Dong and Zhang, 2022, Long *et al.* 2023) or Bayesian frameworks (Zhao *et al.* 2021, Li and Zhou, 2022). While most are designed for single-sample analysis, some also support joint clustering across multiple samples (Dong and Zhang 2022, Singhal *et al.* 2024).

Methods for identifying highly variable genes (HVGs) and differentially expressed genes (DEGs), originally developed for bulk and single-cell transcriptomics, are commonly used for feature selection and cluster characterization (Robinson *et al.* 2010, Brennecke *et al.* 2013, Love *et al.* 2014). However, these methods cannot explicitly incorporate spatial information. In contrast, SVG methods (Svensson *et al.* 2018, Weber *et al.* 2023, Cai *et al.* 2024) leverage spatial coordinates to identify genes with non-uniform expression patterns across the tissue. These spatially-aware approaches provide an alternative to traditional feature selection tools, and can help identify potentially biologically informative genes for further experimental validation.

Most tools for SRT data can only analyze individual samples; among the few exceptions, *DESpace* (Cai *et al.* 2024) supports biological replicates, while *SPADE* (Qin *et al.* 2024) allows comparisons between experimental conditions. Nonetheless, the original version of *DESpace* (Cai *et al.* 2024) does not compare groups of samples, while *SPADE* (Qin *et al.* 2024) is restricted to comparing two individual samples only, and has high computational demands, which makes it impractical for large-scale, multi-sample, multi-condition datasets. As a result, no method can currently compare, from SRT data, spatial patterns across groups of samples.

**Figure 1:**
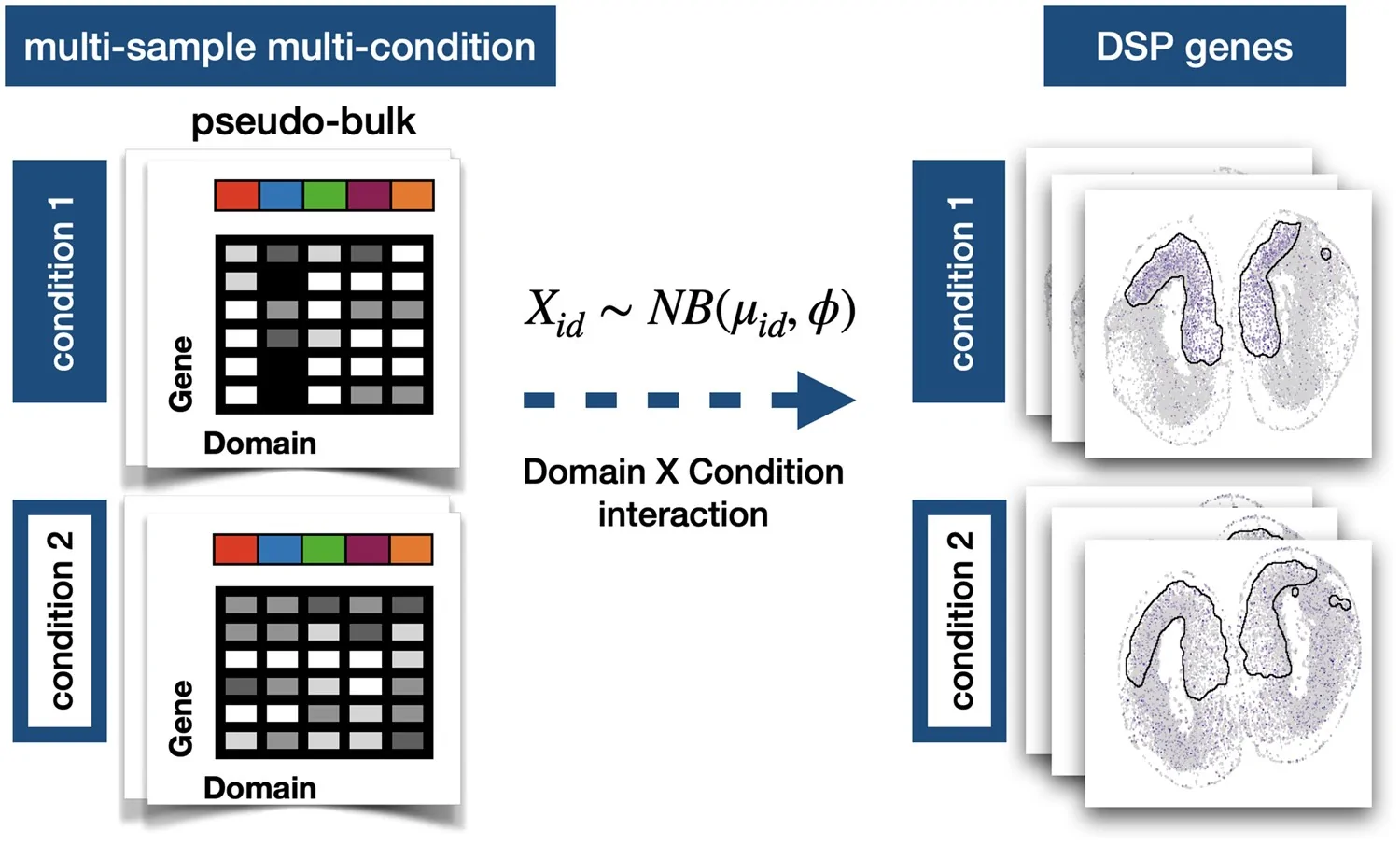
Overview of the *DESpace2* framework for DSP analysis. Given spatial transcriptomics data and spatial domain annotations, the workflow first performs pseudobulking and then conducts differential expression analyses across multiple samples and experimental conditions to identify DSP genes; in the example shown (right panel), a gene has a differential level of expression in one condition versus the other within the same domain.

Here, we introduce a framework to compare spatial patterns from multi-sample, multi-condition SRT data, and identify differential spatial pattern (DSP) genes, i.e. genes whose spatial expression profiles vary between two or more experimental conditions (Fig. 1). A DSP gene may, for example, be spatially variable (SV) in one condition but not in another one, or, alternatively, it may be SV in all conditions but with different spatial patterns. In contrast, genes with uniform abundance across space in all conditions, and SVGs with unchanged spatial structure between conditions, are not considered DSPs. Identifying DSP genes can provide valuable insight into how gene expression patterns vary across experimental conditions, facilitating the understanding of the underlying biological processes.

## 2 Materials and methods

### 2.1 Overview

To detect DSP genes, we expanded our previous framework, *DESpace* (Cai *et al.* 2024), which identifies SVGs using pre-annotated spatial clusters, and fits an *edgeR* (Robinson *et al.* 2010) negative binomial model to the mRNA abundance of each spot, either a single cell or a multi-cellular bin, with spatial clusters as covariates. In this work, we still employ the *edgeR* negative binomial model, but use a more complex design that includes (at least) three covariate sets: spatial domains, conditions, and their interactions. These coefficients capture the average spatial structure of gene expression, condition-specific changes in abundance, and how spatial patterns change between conditions, respectively. This flexible framework also allows additional additive terms to be incorporated to account for potential batch effects or other sample-specific covariates. To make comparisons between the spatial (gene expression) structures of multiple conditions (i.e. DSP), we test whether the interaction terms between conditions and spatial domains differ from zero. Two hypothesis testing frameworks are available: the quasi-likelihood F-test (QLF) and the likelihood ratio test (LRT). By default, our package uses the QLF framework due to its improved error control, by accounting for uncertainty in dispersion estimation.

Our two-step framework shares similarities with DEG and marker gene methods that, although not originally designed for detecting DSP, can be adapted to perform that task. In particular, the *spatialLIBD* R package (Pardo *et al*. 2022) computes pseudobulk counts in each spatial cluster (i.e. adding gene counts from all spots/cells in a cluster), and performs differential testing between clusters using *limma* (Ritchie *et al.* 2015), based on pairwise t-tests or ANOVA F-statistics. To detect DSP, the same strategy can be adapted by testing, in each individual cluster, for differences between experimental conditions, and aggregating results across all clusters (see Supplementary Section S1.1, available as supplementary data at *Bioinformatics* online). Similarly, marker gene methods, such as *scran’*s findMarkers (Lun *et al.* 2016) and *Seurat’*s FindMarkers (Satija *et al.* 2015), designed to compare clusters in individual samples, can also be used to detect DSP by comparing groups of samples in individual clusters, and again aggregating results across clusters (see Supplementary Section S1.1, available as supplementary data at *Bioinformatics* online). However, marker methods treat each spot as an independent replicate, neglecting the dependency between spots from the same sample, which makes them prone to inflated false positive rates (Satija *et al.* 2015, Squair *et al.* 2021, Zimmerman *et al.* 2021).

Although our framework is conceptually related to marker gene methods and *spatialLIBD*, it has a key difference: rather than testing for changes within individual clusters individually (i.e. one test per cluster), we evaluate overall changes across all spatial clusters, capturing global spatial effects in a single test. In addition, our framework can also reveal the specific areas of the tissue affected by DSP, identifying the spatial pattern that displays the biggest difference between conditions. Furthermore, our tool can also compare more than two conditions, and the evolution of spatial patterns across time. Finally, because of its pseudobulk structure, our approach is computationally efficient and is thus compatible with any type of SRT data. Our current approach has been implemented within the pre-existing *DESpace* Bioconductor/R package (from version 2.0.0); below, we will refer to our DSP framework simply as *DESpace2*.

### 2.2 Inference

Consider a SRT dataset with *N* total samples, collected under $N_{c}$ experimental conditions, where each tissue section has been partitioned into *D* spatial domains, and where the *d*-th domain is associated to $N_{d}$ spots. Within each sample, we sum the abundance of all spots belonging to a given domain to compute pseudobulk abundances, i.e. total abundance of a sample within a domain. For a given gene (index omitted for clarity), let $X_{id}$ denote the pseudobulk abundance in sample *i* and domain *d*; we assume that $X_{id}$ is a negative binomial random variable:


(1) $$\begin{array}{c} X_{id}∼NB(\mu_{id},\phi ), \end{array}$$

(2) $$\begin{array}{cc} \;\text{log}(\mu_{id}) & =\text{log}(M_{id})+\beta_{d}+\beta_{c_{i}}+\beta_{c_{i}d},\\ & \;\text{for}\;i=1,\ldots ,N,\;d=1,\ldots ,D,\\ & \;\text{and}\;c_{i}=1,\ldots ,N_{c}, \end{array}$$

Where NB(μ,ϕ) indicates a negative binomial distribution with mean μ and variance μ(1+μϕ); ϕ is the gene-specific dispersion; $M_{id}$ denotes the effective library size for sample *i* in domain *d*, calculated as the domain-specific total count multiplied by the trimmed mean of M values (TMM) normalization factor (Robinson and Oshlack 2010); $\beta_{d}$ represents the coefficient for spatial domain *d*, $\beta_{c_{i}}$ is the coefficient for condition $c_{i}$ to which sample *i* belongs, and $\beta_{c_{i}d}$ captures the interaction between domain *d* and condition $c_{i}$.

We use a global test, to determine whether the spatial structure of gene expression varies between conditions; in particular, we test the null hypothesis that all interaction coefficients are zero:


(3) $$H_{0}:\beta_{c_{i}d}=0,$$

(4) $$\begin{array}{c} \text{for}\;c_{i}=1,\ldots ,N_{c},\;\text{and}\;d=1,\ldots ,D;\\ H_{1}:\;\text{otherwise} \end{array}$$

This system of hypotheses can be tested using either a LRT (Wilks 1938) or a QLF (Lund *et al.* 2012, Chen *et al.* 2025). Both tests use the same negative binomial generalized linear model defined in (1), but differ in how dispersion uncertainty is incorporated in the inference. The LRT is based on the negative binomial log-likelihood with fixed dispersion ϕ, which often results in a more sensitive but potentially liberal test. In contrast, the QLF framework uses gene-specific quasi-likelihood dispersions together with empirical Bayes shrinkage, thereby accounting for uncertainty in dispersion estimation. This typically yields more conservative and better calibrated inference, especially with limited sample sizes or heterogeneous gene-wise variability.

We also employ an individual-domain test to pinpoint the main DSP region, i.e. the domain where spatial patterns vary the most across conditions. For each domain *d*, we relabel the data into two categories, domain *d* and all remaining domains combined, and fit a negative binomial model with relabeled domain, condition, and their interaction as covariates. We then test, via a QLF or LRT, the interaction term between the relabeled domain and condition (see Supplementary Section S1.2, available as supplementary data at *Bioinformatics* online).

In all analyses, model fitting and differential testing use *edgeR* (Robinson *et al.* 2010, McCarthy *et al.* 2012, Chen *et al.* 2016), while Benjamini-Hochberg *P*value correction (Benjamini and Hochberg 1995) is applied to account for multiple testing, and control for the false discovery rate (FDR).

### 2.3 Simulation studies—anchor data

To evaluate our approach, we designed benchmarks utilizing real and semi-simulated data based on two SRT datasets, referred to as *LIBD* (Maynard *et al.* 2021) and *ARTISTA* (Wei *et al.* 2022).

The *LIBD* (Maynard *et al.* 2021) dataset, obtained from the Visium platform (Ståhl *et al.* 2016), contains 12 samples from three human brain donors. Pathologists annotated each sample into white matter and up to six cortical layers (Fig. S1, available as supplementary data at *Bioinformatics* online). Each sample contains, on average, 33 538 genes and 3973 spots; after filtering (Supplementary Section S1.3, available as supplementary data at *Bioinformatics* online), 14 628 genes across 3936 spots were retained (Table S1, available as supplementary data at *Bioinformatics* online). The number of genes shared across samples was 33 538 before filtering and 13 743 after filtering.

The *ARTISTA* (Wei *et al.* 2022) dataset, obtained from Stereo-seq (Chen *et al.* 2022), captures axolotl telencephalon regeneration at single-cell resolution. The study aims to investigate brain healing after injury, and includes tissue samples from five distinct regeneration stages (days post-injury: DPI), with three to four sections collected from each stage, resulting in 16 samples in total. In the absence of pre-annotated spatial patterns, we applied *Banksy* multi-sample clustering to group cells into five distinct spatial clusters, which are consistent across samples (Fig. S2, available as supplementary data at *Bioinformatics* online). On average, each sample includes measurements for 27 077 genes across 9389 cells (Table S1, available as supplementary data at *Bioinformatics* online). After filtering (Supplementary Section S1.3, available as supplementary data at *Bioinformatics* online), we retained 13 890 genes common to all samples and an average of 9215 cells per sample.

### 2.4 Simulation studies—SV profiles

In order to generate realistic data, we used the *LIBD* and *ARTISTA* datasets as anchors to generate several *semi-simulated* datasets. In each simulated dataset, we allocated replicate samples to two artificial conditions. Specifically, for the *LIBD* data, one group includes one slice from each of the three donors, while the other condition includes a different slice from the same donors. For the *ARTISTA* data, we used eight tissue sections from four regeneration stages (2, 5, 10, and 20 DPI), and randomly assigned one section of each stage to one condition, using the remaining section for the other condition.

Using pre-annotated spatial domains (i.e. pathologist annotations for the *LIBD* data, and *Banksy* clusters for the *ARTISTA* data; see Supplementary Figs. 1 and 2, available as supplementary data at *Bioinformatics* online), we generated a variety of spatial patterns by re-allocating real measurements across the tissue in different ways (see Supplementary Section S1.4, available as supplementary data at *Bioinformatics* online). In particular, we obtained uniform as well as SV patterns that correspond to real structures, e.g. the brain cortex layers in the *LIBD* dataset.

Uniform structures were generated by randomly permuting observations across the entire tissue, while spatial structures were created by randomly selecting a spatial domain to have higher (main text results) or lower abundance (Fig. S3, available as supplementary data at *Bioinformatics* online) compared to the rest of the tissue; examples are shown in Supplementary Figs. 4 and 5, available as supplementary data at *Bioinformatics* online.

**Figure 2:**
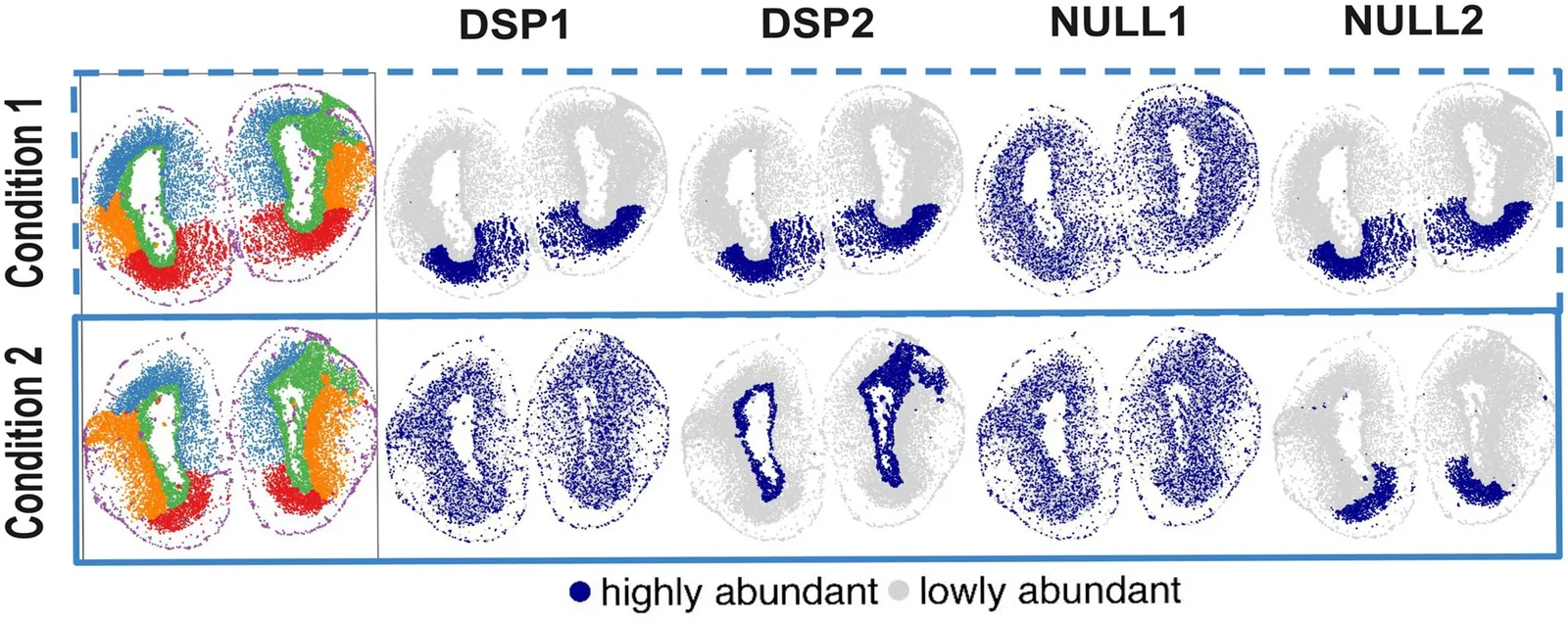
Simulation design of the four simulated categories: *DSP1*, *DSP2*, *NULL1*, and *NULL2* using the *ARTISTA* dataset. The first column shows *Banksy* spatial domains, and the next four columns present example of simulations for each category. Two rows correspond to different conditions.

In this way, we generated four distinct scenarios, *DSP1*, *DSP2*, *NULL1*, and *NULL2* (Fig. 2), and randomly assigned genes to each in equal proportions (25% per scenario). In the DSP scenarios, gene expression patterns differ between conditions: *DSP1* genes are highly (or lowly) abundant in a randomly selected spatial domain in one condition (selected at random), and have uniform expression in the other condition; *DSP2* genes are SVGs in both conditions but with different spatial expression structures in the two conditions (two distinct randomly selected spatial domains). In contrast, the NULL scenarios have identical spatial structures in both conditions: *NULL1* genes are spatially uniformly expressed in both conditions, while *NULL2* genes are SVGs sharing the same spatial pattern in both conditions (again, with a randomly selected spatial domain). The consistent spatial structure of *NULL2* genes in the same clusters across samples facilitates accurate detection of spatial domains when combining the four distinct scenarios.

## 3 Results

### 3.1 Simulation studies—global test

In the *LIBD* and *ARTISTA* simulated datasets, we re-estimated the spatial patterns via *BayesSpace* and *Banksy*, respectively, leading to five consistent spatial domains per sample (Supplementary Section S1.5, available as supplementary data at *Bioinformatics* online). All methods were evaluated using these (noisy) estimated clusters rather than the true cluster labels. The simulated data were analyzed via *DESpace2* (DSP functionality with QLF and LRT testing), *scran’*s findMarkers (Lun *et al.* 2016), *Seurat’*s FindMarkers (Satija *et al.* 2015) (both in the single-cell and pseudobulk versions), and *spatialLIBD’s wrapper* (Pardo *et al*. 2022). Since no existing approach is designed to detect changes in the spatial structure of gene expression, between groups of samples, we adapted these methods for DSP detection. We excluded *SPADE* (Qin *et al.* 2024) as it compares individual samples only and is computationally intensive: in our tests, it required approximately two hours to fit a single gene with approximately 4000 spots.

**Figure 3:**
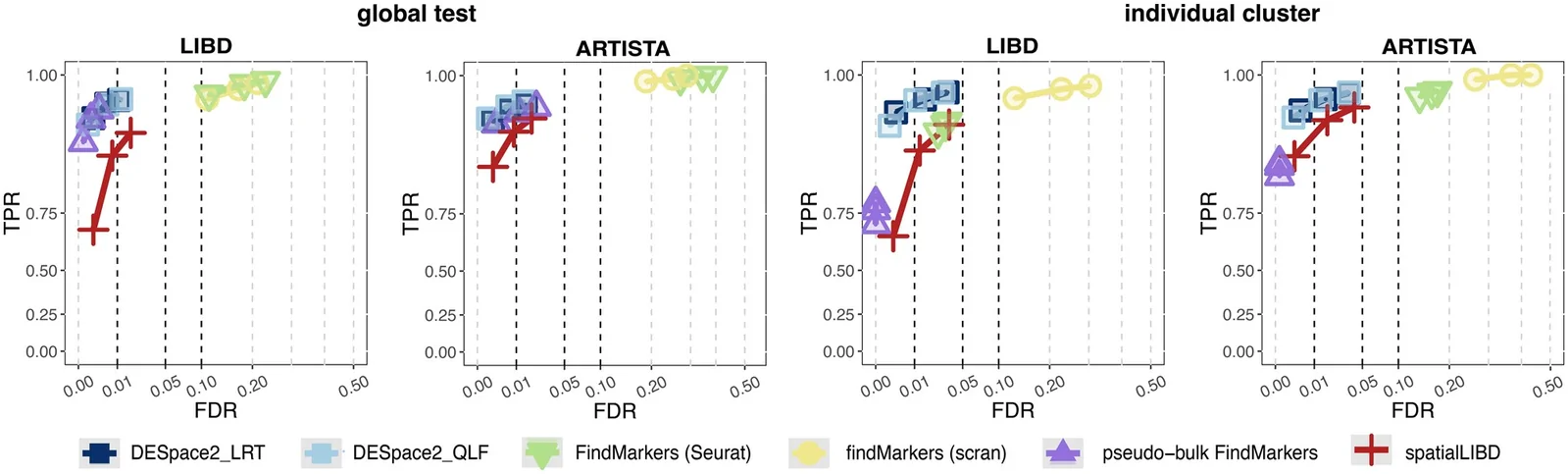
TPR *vs*. FDR for DSP gene detections in simulations. The left two panels correspond to the global test (*LIBD* and *ARTISTA* datasets), and the right two panels to the individual-domain test. Points show observed TPR and FDR at nominal thresholds of 0.01, 0.05, and 0.1.

The left two panels of Fig. 3 show the true positive rate (TPR) *vs.* false discovery rate (FDR) across all four simulation scenarios (i.e. *DSP1*, *DSP2*, *NULL1* and *NULL2*) combined. Across our semi-simulated benchmarks, *DESpace2* controls the FDR and offers competitive statistical power. findMarkers and FindMarkers also achieve a good TPR, but exhibit an inflated FDR, leading to a significant number of false discoveries. Conversely, pseudobulk-based FindMarkers and *spatialLIBD*, control the FDR, but have lower statistical power, possibly due to the fact that they compare pairs of clusters rather than jointly capturing global changes across all clusters, as *DESpace2* does.

We also compared *DESpace2* results using spatial domains re-estimated by *BayesSpace* or *Banksy* against the ground-truth domains used for simulation, and observed only minor differences, likely because the recomputed clusters closely matched the ground truth (Fig. S6, available as supplementary data at *Bioinformatics* online).

Additionally, we investigated FDR; to this end, we analyzed the distribution of raw *P*value for NULL1 and NULL2 genes (i.e. genes with uniform expression or identical spatial patterns across conditions). In all scenarios, *DESpace2* shows no inflation of false positives, while all our competitors display inflated false positive rates (Fig. S7, available as supplementary data at *Bioinformatics* online); this is likely because our competitors perform a test for each spatial pattern, and the global score is obtained as the minimum of the individual cluster *P*value. Indeed, as expected, the individual-domain *P*value are more conservative (see next Section).

Figure S3, available as supplementary data at *Bioinformatics* online presents the simulation results where the selected spatial domain has lower abundance than the rest of the tissue. Here, the spatial signal is diluted by the broad high-abundance region (comprising the D−1 domains), complicating DSP detection. As above, all methods yield lower TPRs, and their ranking remains stable.

We further evaluated robustness to clustering perturbations, including mis-clustering and variations in clustering resolution. Across these scenarios, *DESpace2* maintains well-calibrated FDR while retaining higher or comparable power relative to competing methods (Supplementary Section S1.6, available as supplementary data at *Bioinformatics* online and Figs. 8–12). We also investigated performance when the DSP signal is weaker: all methods exhibited lower TPRs, while their relative ranking remained consistent across signal strengths. Finally, within *DESpace2*, we also tried to replace *edgeR* differential framework with the one implemented in *DESeq2*: results are similar, with *edgeR*-based *DESpace2* being slightly faster (Supplementary Section S1.6, available as supplementary data at *Bioinformatics* online and Figs. 14–16).

### 3.2 Simulation studies—individual-domain

We also evaluated *DESpace2*’s ability to identify the domains where gene abundance varies across conditions. The individual-domain test requires at least three clusters (with two it is equivalent to the global test), and may be particularly useful in datasets with many clusters, as it may help biologists to focus on specific domains of interest.

Using the same simulation framework as in the global test, we assessed whether methods correctly identify the main pattern that varies between conditions. *DSP1* genes are SV in one condition only: the key DSP cluster is the one selected to have higher or lower abundance in the SV condition. *DSP2* genes are SVGs under both conditions, but with different spatial structures; the two main DSP clusters are the two clusters with higher or lower abundance in each condition (Fig. 2).

For each method, we tested all domains, and counted how often the most significant domain(s) correspond to the main DSP cluster (for *DSP1* genes), or the main two DSP clusters (for *DSP2* genes). For *DSP1* genes, *DESpace2* identified the main DSP cluster in 96–100% of genes; FindMarkers and findMarkers led to similar percentages, while *pseudobulk*FindMarkers and *spatialLIBD* displayed lower accuracy. For *DSP2* genes, *DESpace2* using the QLF identified the two main DSP clusters in 99.9 (96.0) and 100 (98.4)% of genes in our semi-simulated *LIBD* and *ARTISTA* datasets, respectively, when the main clusters had higher or lower abundance, with competitors showing comparable performance (Supplementary Tables 2 and 3, available as supplementary data at *Bioinformatics* online).

We also generated TPR *vs.* FDR plots (right two panels of Fig. 3). In this case, all domains are DSP for DSP genes, although most show only minor changes between conditions. Therefore, in DSP genes, we only considered the main spatial clusters involved (i.e. the main cluster for *DSP1*, and the two main clusters for *DSP2*); as controls, we included all clusters from NULL patterns. As in the global test, *DESpace2* has good FDR control, and higher TPR than competitors. Notably, the LRT version of *DESpace2* achieves higher sensitivity than its QLF counterpart under this setting.

We also plotted the densities of NULL1 and NULL2 *P*value (Supplementary Figs. 17 and 18, available as supplementary data at *Bioinformatics* online): all methods provide approximately uniform (or slightly conservative) null distributions, except for FindMarkers and findMarkers, where a general inflation toward 0 is observed, indicating increased false positive rates.

With a low-abundance main domain, all methods similarly lost power due to the subtler DSP structure (Supplementary Figs. 3 and 5, available as supplementary data at *Bioinformatics* online).

Sensitivity analyses across clustering perturbations, varying spatial signals, and an alternative *DESeq2*-based implementation of *DESpace2* yielded consistent results (Table S4, available as supplementary data at *Bioinformatics* online and Figs. 8–16).

### 3.3 Applications to real data

We applied all methods to the original *ARTISTA* dataset, comparing two conditions (2 and 20 DPI), which are the earliest and latest replicated time points, and may represent the most distinct groups for analysis. Additionally, for *DESpace2* and *spatialLIBD*, which both support comparisons across more than two conditions, we also included all five time points (2, 5, 10, 15, and 20 DPI), each with three or four samples (Table S1, available as supplementary data at *Bioinformatics* online). In the absence of manually annotated clusters, spatial clusters were computed by jointly applying *Banksy* to all 16 samples across five time points. For the two-group comparison, the data were then subset to 2 and 20 DPI, ensuring that both two- and five-group comparisons used the same spatial domain annotations and number of genes. For the two-condition analysis, *DESpace2* and *spatialLIBD* identified only tens to a few hundred significant genes, whereas FindMarkers and findMarkers reported over 7000 out of 13 890 genes as significant. This large discrepancy is consistent with the inflation of false positives observed in simulation studies (Table S5, available as supplementary data at *Bioinformatics* online).

Unlike simulations, real data do not have a ground truth. We therefore used two curated gene lists as external biological references, retrieving “GOBP_WOUND_HEALING” and “GOBP_REGENERATION” from the Molecular Signatures Database (MSigDB v2024.1.Hs) (Subramanian *et al.* 2005, Liberzon *et al.* 2011, 2015). Because axolotl-specific functional curation is limited, these human-curated MSigDB sets served as proxies for biological relevance. We then selected the top 200 (i.e. most significant) genes from each method, and counted how many appeared in the two lists. We also computed their average rank, where a rank of 1 indicates the most significant gene returned by a method. The top 200 genes from *DESpace2*, in both its 2-condition and 5-condition comparisons, contained more genes from the wound healing and regeneration lists than those from competitors, and those genes had lower average ranks (i.e. were more significant) (Table S5, available as supplementary data at *Bioinformatics* online). We also applied *DESpace2* with a spline model to account for the effect of time (i.e. spline-based *DESpace2*), which, despite recovering fewer interesting genes among the top 200, achieved a lower average rank.

Furthermore, we retrieved healing- and regeneration-related genes from The Human Protein Atlas website (Pontén *et al.* 2008). The results were consistent with the MSigDB-based analysis: *DESpace2* detected either more or the same number of healing- and regeneration-related genes as competing methods, and these genes had lower average ranks (Table S6, available as supplementary data at *Bioinformatics* online).

To further assess the functional relevance, we performed over-representation analysis of genes identified by each method. Among the top 200 returned Gene Ontology terms, *DESpace2* recovered more terms related to “wound healing” or “regeneration” than competing methods (Table S7, available as supplementary data at *Bioinformatics* online).

Finally, we investigated the computational efficiency of each method by measuring runtime on the *ARTISTA* dataset (Supplementary Figs. 19 and 20, available as supplementary data at *Bioinformatics* online). Methods based on pseudobulk counts, including *spatialLIBD*, *DESpace2*, and pseudobulk FindMarkers, were faster than spot-level approaches such as *scran’*s findMarkers and *Seurat’*s FindMarkers. For *DESpace2*, the runtime was 14 seconds for the global test and 36 seconds for individual tests using the QLF implementation (*DESpace2_QLF*); runtimes for *DESpace2_LRT* were similar (14 and 38 seconds, respectively).

All methods rely on precomputed spatial clusters; therefore, runtimes reflect only the differential testing step. For reference, spatial clustering with *Banksy* required 97 minutes for the 5-group comparison (16 samples).

## 4 Discussion

We presented a novel framework, based on spatial domains and DEG testing, to identify DSP genes, i.e. genes where structure of spatial expression varies between groups of samples, which represents a type of analysis that is absent from the current spatial omics toolbox.

We benchmarked our approach against diverse alternatives (e.g. adapting marker and DE methods) in *ad hoc* semi-simulated and real data analyses, where our framework demonstrates good statistical power (i.e. TPR), controls for the FDR, and is computationally efficient. Our method can detect differences across the entire tissue (i.e. global test), or test each specific cluster (i.e. individual-pattern test), identifying the main region that varies across conditions. Our tool accepts flexible, user-customizable design matrices; this allows users, for instance, to explicitly account for batch effects across samples, compare more than two conditions, and model time courses (e.g. via splines). Users can choose between the QLF- and LRT-based testing options, depending on their needs: QLF generally provides more stable error control and is used as the package default, whereas LRT can offer increased sensitivity when the spatial signal is diluted.

Our DSP framework is freely distributed within the *DESpace* Bioconductor R package, which simplifies its usage and integration with other pipelines, and is accompanied by an example usage DSP vignette and *ad hoc* plotting functions. Furthermore, our approach is flexible and can be applied to any spatial transcriptomics technology.

We also acknowledge some limitations of our work. Our approach relies on predefined spatial clusters, which is generally manageable in practice: spatial clustering is a standard step in spatial transcriptomics analysis, with over 50 spatial clustering methods developed since 2020 (Moses and Pachter 2022). Importantly, because of this dependence, our method is more sensitive to detecting DSP genes whose expression aligns with the spatial patterns provided, having lower statistical power to detect genes that do not follow these structures (found to be more rare in previous research (Weber *et al.* 2023, Cai *et al.* 2024)). Additionally, domains that exclusively appear in one condition cannot be analyzed; nonetheless, these domains indicate a condition-specific region (i.e. present in some conditions and absent in others), rather than a DSP region. This is conceptually similar to what happens with differential state analyses (Weber *et al.* 2019, Crowell *et al.* 2020, Tiberi *et al.* 2023), which target differences in abundance only in cell clusters that appear in all conditions, and ignore differences in cell cluster proportions.

In some cases, DSP could represent shifts in composition of the cell types within spatial clusters. Such changes may still be of direct interest, or could be adjusted for as covariates.

Overall, we believe that our DSP framework represents a useful instrument for investigating spatial omics data; additionally, it also represents an initial step for comparing groups of samples from SRT data, and may pave the road for other future methods in the field.

## Supplementary Material

btag450_Supplementary_Data

## Data Availability

Our DSP framework is implemented in *DESpace* Bioconductor R package (from version 2.0.0). Scripts and results are available on GitHub (https://github.com/peicai/DESpace2_manuscript) and Zenodo (DOI: 10.5281/zenodo.20206228). The *LIBD* (Maynard *et al.* 2021) dataset was obtained from the *spatialLIBD* (Pardo *et al*. 2022) R Bioconductor package, and the original *ARTISTA* (Wei *et al.* 2022) dataset is available at https://db.cngb.org/stomics/artista/download/. Processed *ARTISTA* data are provided via the *muSpaData* ExperimentHub R Bioconductor package.
