# Supplementary material for: DESpace2: detection of differential spatial patterns in spatial omics data
Source: Bioinformatics. 2026 Aug 21;42(Suppl 2):btag450. doi: 10.1093/bioinformatics/btag450 (PMC13501297; doi:10.1093/bioinformatics/btag450)
Supplement: Supplementary file 1 [file btag450_supplementary_data.pdf]

SUPPLEMENTARY MATERIAL
DESpace2: detection of differential spatial patterns in spatial omics data

Peiying Cai[1*], Mark D Robinson[1*], and Simone Tiberi[1,2*]

[1] *Department of Molecular Life Sciences and Swiss Institute of Bioinformatics, University of Zurich, Zurich, Switzerland.*
[2] *Department of Statistical Sciences, University of Bologna, Bologna, Italy.*
[*] e-mail: Peiying.Cai@uzh.ch, Mark.Robinson@mls.uzh.ch, Simone.Tiberi@unibo.it

1 Supplementary Details

1.1 Adjusted p-values

For all methods, we controlled the false discovery rate using Benjamini–Hochberg adjusted p-values [Benjamini and Hochberg, 1995].

For *spatialLIBD* [Pardo et al.], *scran*'s `findMarkers` [Lun et al., 2016], *Seurat*'s `FindMarkers`, and pseudobulk-based `FindMarkers` [Satija et al., 2015], we computed per-gene q-values using the `perGeneQValue` function from *DEXSeq* [Anders et al., 2012, Love et al., 2014]. This was done to summarize and correct for multiple testing across clusters.

1.2 Design matrix for the individual-domain test

For the individual-domain test, each spatial domain is evaluated separately. For a given domain d, the data are relabeled into two categories: the target domain d, denoted as "Target", and all remaining domains combined, denoted as "Other". For a given gene (index omitted for clarity), let $Z_{ir}^{(d)}$ denote the pseudobulk abundance for sample i and relabeled category $r \in \{\text{Target}, \text{Other}\}$. Using the same negative binomial parameterization as in the global test, we assume

$$Z_{ir}^{(d)} \sim NB(\mu_{ir}^{(d)}, \phi),$$

with

$$\log(\mu_{ir}^{(d)}) = \log(M_{ir}^{(d)}) + \alpha_r^{(d)} + \alpha_{c_i}^{(d)} + \alpha_{c_i r}^{(d)}.$$

Here, $M_{ir}^{(d)}$ denotes the effective library size, $\alpha_r^{(d)}$ represents the coefficient for the relabeled domain, $\alpha_{c_i}^{(d)}$ is the coefficient for the condition to which sample i belongs, and $\alpha_{c_i r}^{(d)}$ captures the interaction between condition and relabeled domain.

The model is parameterized using reference levels, with one condition and "Other" chosen as the reference levels for condition and relabeled domain category, respectively, and interaction terms defined relative to these baselines. The resulting design matrix is required to be full rank, and the library size term $\log(M_{ir}^{(d)})$ is treated as a known offset.

For each domain d, we test whether the interaction terms associated with the target domain are zero:

$$H_0 : \alpha_{c,\text{Target}}^{(d)} = 0, \quad \text{for all non-reference conditions } c,$$

against the alternative that at least one such interaction coefficient is nonzero. This procedure is repeated for all domains.

1.3 Quality control and filtering

Quality control procedures were performed according to the guidelines outlined in the online book "Orchestrating Spatially-Resolved Transcriptomics Analysis with Bioconductor" [Amezquita et al., 2020].

To identify low-quality spots or cells, we used the *Scuttle*'s `addPerCellQC` function [McCarthy et al., 2017]. Spots/cells were evaluated based on total UMI counts, the number of detected genes (i.e., genes with non-zero UMI counts), the proportion of reads mapped to mitochondrial genes, and the estimated number of cells per spot. We then applied sample-specific thresholds to filter out low-quality spots/cells.

We also removed undetected or lowly expressed genes, retaining only those expressed in at least 20 non-zero spots/cells for downstream analysis.

Finally, to enable cross-sample analysis, we excluded genes that were not present in all samples.

1.4 Simulating spatially variable (SV) patterns

To generate simulated data, we first partitioned each tissue sample into two regions, R_1 and R_2, containing n_1 and n_2 spots or cells, respectively. We then adopted the `runPatternSimulation` function from *Giotto*'s R package [Dries et al., 2021] to assign gene expression patterns based on these regions. For each selected gene, the function redistributes expression measurements across spots/cells, with highly expressed values assigned to region R_1 with probability π. Accordingly, $\pi = 1$ assigns the n_1 highest expression values to R_1, creating a strong spatial pattern, whereas $\pi = 0.5$ results in uniform expression across both regions.

Next, we simulated a mixture of uniformly expressed and spatially variable genes (SVGs). Uniform patterns were generated by sampling π from a Beta distribution with a mean of 0.5 and a standard deviation of 0.025. In contrast, SVGs were modeled using a Beta distribution with a mean of 0.9 and the same standard deviation. To evaluate robustness to weaker spatial signals, we additionally considered SVG simulations with Beta distributions centered at 0.8 and 0.7, while keeping the same standard deviation. Representative examples of simulated patterns across signal strengths are shown in Supplementary Figure 14. For each SVG, we randomly selected a spatial domain to serve as R_1 and assigned the most abundant expression measurements to this region, using randomly sampled π. This approach ensured that the selected domain exhibited higher average expression compared to the rest of the tissue. Using the uniform and/or spatial patterns generated for each sample under each condition, we created four categories of possible scenarios - *DSP1*, *DSP2*, *NULL1*, and *NULL2* - and randomly assigned genes to each scenario in equal proportions (25% each).

We also generated a parallel simulation in which SVGs were lowly abundant in the selected domain(s), instead of highly abundant. This allowed us to evaluate method performance under both high- and low-abundance spatial expression settings.

1.5 Spatial domains used in simulation

For the *LIBD* dataset, we used pathologist-provided annotations from the original dataMaynard et al. [2021] to define spatial patterns for simulation. To harmonize the number of spatial domains across samples, we reassigned "Layer1" and "Layer2" to "Layer6" in samples where they were present, yielding five common domains (Layers 3–6 and WM) for cross-sample simulation. After generating the simulated data, spatial domains were re-estimated for each sample using *BayesSpace*. Given the clear structure of cortical layers and white matter, manual alignment of spatial domains across samples was straightforward.

In contrast, the *ARTISTA* dataset lacked predefined spatial annotations. To define consistent spatial domains across samples, we applied joint clustering using *Banksy*. Simulations were then performed based on these domains. Afterward, we re-estimated the spatial domains using *Banksy*, jointly across all samples, to provide input for each method.

1.6 Sensitivity analyses

Since our framework relies on predefined spatial domains, we further assessed each method's robustness under three perturbation settings: mis-clustering, clustering resolution and weaker DSP signal.

For mis-clustering, three scenarios were examined. First, a random subset of cells (1%, 5%, 10%, and 20%) was randomly reassigned to incorrect clusters (Supplementary Figure 8). Second, to mimic more realistic spatially localized domain-detection errors, we identified cells near domain boundaries and reassigned a subset of them to neighboring (incorrect) clusters (Supplementary Figure 8). Boundary cells were defined using local neighborhood information, and reassignment probabilities were weighted by proximity to neighboring domains. Third, we simulated datasets using five spatial domains, and forced *Banksy* to recover an incorrect number of clusters (varying from three to ten). As the proportion of randomly mis-assigned cells increases, all methods show reduced true positive rates (TPRs) (Supplementary Figure 9); nonetheless, *DESpace2* consistently achieves higher TPRs than competing approaches, while controlling the false discovery rate (FDR) in both global and individual-domain tests. Boundary-localized mis-clustering produced similar results, with small TPR reductions but consistent relative method rankings and FDR control (Supplementary Figure 10). Similarly, when the number of clusters was mis-specified, all methods exhibit reduced performance, particularly when the number of clusters was underestimated (Supplementary Figure 11). Instead, when the number of sub-clusters is overestimated, all methods show smaller declines in TPR; importantly, across the evaluated scenarios, *DESpace2* maintains a well-calibrated FDR. In this setting, the quasi-likelihood F-test (QLF) version of *DESpace2* is more stable than the likelihood ratio test (LRT) version; this is consistent with the more conservative inference by quasi-likelihood dispersion modeling and empirical Bayes moderation under model misspecification.

Next, we evaluated robustness to clustering resolution by simulating datasets with varying numbers of clusters (Supplementary Figures 12 and 13). In the global test, *DESpace2_ QLF* has the highest TPR with two, four, and six clusters, and remains comparable to the pseudobulk-based `FindMarkers` at higher resolutions (Supplementary Figure 12). The LRT version follows a similar trend at low resolutions but shows reduced detection power and a marginal increase in FDR as the number of clusters grows. This trend likely reflects the increasing number of coefficients that the LRT needs to estimate in the global test as the clustering resolution increases, making it sensitive to model complexity. In the individual test, *DESpace2* consistently achieves higher TPRs than competitors, particularly with fewer clusters (Supplementary Figure 13). Clustering resolution has a marginal impact on the FDR for both the QLF and LRT implementations. Overall, these results highlight the robustness of *DESpace2*, particularly under the QLF-based test, which, in our benchmarks, shows well-calibrated error rates while maintaining good power.

We further assessed performance robustness under weaker spatial signals, generated from Beta distributions centered at 0.8 and 0.7. As expected, statistical power decreased with weaker signals, while the relative ranking of methods remained consistent (Supplementary Figures 14 and 15). Detection of the main SV domains followed a similar trend (Supplementary Table 4). Under intermediate to strong spatial signals, most methods accurately identified the main SV domain(s), correctly detecting over 95% of *DSP1* genes and nearly all *DSP2* genes. At the weakest signal, performance dropped across methods, especially for *DSP1*, whereas *DESpace2* remained among the top-performing approaches.

Finally, to assess sensitivity to the underlying differential expression implementation, we compared *DESpace2* based on *edgeR* [Robinson et al., 2010] to *DESpace2* based on *DESeq2* [Love et al., 2014]. The *DESeq2* implementation of *DESpace2* uses a LRT comparing the full model including condition-by-domain interactions to a reduced model without interaction terms. Performance was comparable across implementations, with *edgeR* implementation of *DESpace2* being slightly faster (Supplementary Figure 16).

1.7 Software versions

All analyses were performed in R (version 4.4.2, with Bioconductor packages from release 3.20).

2 Supplementary Tables

Dataset	Sample ids	Before filtering		After filtering	
		# of genes	# of spots/cells	# of genes	# of spots/cells
LIBD	151507	33538	4226	14226	4172
	151508	33538	4384	13835	4333
	151509	33538	4789	14561	4738
	151510	33538	4634	14331	4606
	151669	33538	3661	14547	3647
	151670	33538	3498	14183	3473
	151671	33538	4110	14926	4079
	151672	33538	4015	14634	3989
	151673	33538	3639	15115	3604
	151674	33538	3673	15878	3634
	151675	33538	3592	14626	3556
	151676	33538	3460	14672	3405
	Average	33538	3973	14628	3936
ARTISTA	2DPI_1	27324	7668	15520	7544
	2DPI_2	28365	8192	15633	8054
	2DPI_3	28371	8738	15734	8602
	5DPI_1	28741	8106	15995	7947
	5DPI_2	29442	8178	16221	8023
	5DPI_3	29341	8372	16213	8220
	10DPI_1	27600	9440	15623	9253
	10DPI_2	28602	9600	15932	9407
	10DPI_3	27398	10002	15421	9806
	15DPI_1	23331	8996	16289	8817
	15DPI_2	23885	9639	16548	9444
	15DPI_3	22297	9676	15714	9482
	15DPI_4	23271	10792	16404	10575
	20DPI_1	28205	10462	15892	10284
	20DPI_2	28987	11319	16389	11118
	20DPI_3	28069	11048	15822	10856
	Average	27077	9389	15959	9215

Supplementary Table 1: Number of genes and spots for each sample, before and after filtering.

	Method	*LIBD*	*ARTISTA*
DSP1	*DESpace2_LRT*	98.1	99.3
	DESpace2_QLF	96.6	99.5
	Seurat's `FindMarkers`	94.4	98.8
	scran's `findMarkers`	94.1	98.8
	pseudobulk-based `Findmarkers`	85.3	88.9
	spatialLIBD	71.5	80.4
DSP2	*DESpace2_LRT*	100	100
	DESpace2_QLF	99.9	100
	Seurat's `FindMarkers`	99.7	100
	scran's `findMarkers`	99.7	100
	pseudobulk-based `Findmarkers`	99.6	99.6
	spatialLIBD	96.2	99.7

Supplementary Table 2: Individual cluster results when the main spatial cluster has higher abundance than the rest of the tissue. Percentage of times the method identified the main SV cluster in *DSP1* and when the top two detected domains both corresponded to the main SV clusters in *DSP2*, using *BayesSpace* for *LIBD* and *Banksy* for *ARTISTA*.

	Method	*LIBD*	*ARTISTA*
DSP1	*DESpace2_LRT*	97.1	98.4
	DESpace2_QLF	96.9	98.3
	Seurat's `FindMarkers`	94.5	97.4
	scran's `findMarkers`	94.2	97.6
	pseudobulk-based `Findmarkers`	94.7	96.3
	spatialLIBD	92.5	95.5
DSP2	*DESpace2_LRT*	96.8	98.4
	DESpace2_QLF	96.0	98.4
	Seurat's `FindMarkers`	94.9	99.0
	scran's `findMarkers`	94.5	98.6
	pseudobulk-based `Findmarkers`	95.8	97.4
	spatialLIBD	92.9	97.0

Supplementary Table 3: Individual cluster results when the main spatial cluster has lower abundance than the rest of the tissue. Percentage of times the method identified the main SV cluster in *DSP1* and when the top two detected domains both corresponded to the main SV clusters in *DSP2*, using *BayesSpace* for *LIBD* and *Banksy* for *ARTISTA*.

	Method	*ARTISTA*		
		0.7	0.8	0.9
DSP1	*DESpace2_LRT*	92	97.9	99.3
	DESpace2_QLF	93.1	98.2	99.5
	Seurat's `FindMarkers`	84.2	94.4	98.8
	scran's `findMarkers`	90.5	96.6	98.8
	pseudobulk-based `Findmarkers`	86.4	94.6	88.9
	spatialLIBD	78.9	85.2	80.4
DSP2	*DESpace2_LRT*	96.9	99.7	100
	DESpace2_QLF	97.4	99.8	100
	Seurat's `FindMarkers`	93.4	99.3	100
	scran's `findMarkers`	97.1	99.8	100
	pseudobulk-based `Findmarkers`	95.2	99.3	99.6
	spatialLIBD	91.6	98.4	99.7

Supplementary Table 4: Individual cluster results. Results are shown across increasing spatial signal strengths, with spatial strength values of 0.7, 0.8, and 0.9. Percentage of times the method identified the main SV cluster in *DSP1* and when the top two detected domains both corresponded to the main SV clusters in *DSP2*, using *Banksy* for *ARTISTA*.

Method	Identified in top 200			Average rank		# of genes
	Regeneration	Wound healing	Overall	Regeneration	Wound healing	
DESpace2_ QLF (5 groups)	5	15	18	2629	2914	848
DESpace2_ LRT	5	12	16	1943	2016	229
DESpace2_ QLF	4	12	16	1458	1599	71
DESpace2_ LRT (5 groups)	5	12	15	3185	3452	1658
DESpace2_ QLF (individual test)	5	10	14	4386	4613	23
DESpace2_ LRT (individual test; 5 groups)	3	10	13	4979	5318	1322
DESpace2_ QLF (individual test; 5 groups)	3	10	13	4765	5371	372
Seurat's `FindMarkers`	7	6	12	4054	5100	7809
spatialLIBD	7	6	12	5865	6675	289
DESpace2_ LRT (individual test)	1	10	11	3574	3650	152
spline-based *DESpace2_ QLF* (5 groups)	5	6	10	1402	1534	130
scran's `findMarkers`	6	5	10	4122	5159	7016
scran's `findMarkers` (5 groups)	5	5	10	3703	4691	8732
spline-based *DESpace2_ LRT* (5 groups)	4	6	9	1588	1714	261
pseudobulk-based *Seurat*'s `FindMarkers`	6	5	9	5277	6176	2957
spatialLIBD (5 groups)	0	2	2	5994	6526	6544

Supplementary Table 5: Summary of genes detected by each method in the *ARTISTA* dataset. The table shows the number of genes detected, among the top 200 results returned by each method. Gene sets were retrieved from the Molecular Signatures Database (MSigDB v2024.1.Hs) [Liberzon et al., 2011, 2015] for the following keywords: "Wound healing" and "Regeneration". These sets include 434 and 203 genes, respectively, of which 249 and 123 were present in our dataset. "Overall" represents the union of both sets (603 genes total; 354 retained). The table also reports the average rank of the genes in each list (lower values indicate higher significance), and the total number of genes called significant by each method.

Method	Identified in top 200			Average rank		# of genes
	Regeneration	Wound healing	Overall	Regeneration	Wound healing	
DESpace2_LRT	6	9	14	1898	2120	229
DESpace2_QLF	5	10	14	1435	1703	71
DESpace2_QLF (5 groups)	6	9	13	2669	3017	848
spatialLIBD	8	7	13	5949	6862	289
DESpace2_QLF (individual test)	6	8	13	4334	4813	23
DESpace2_LRT (5 groups)	6	6	11	3242	3598	1658
Seurat's `FindMarkers`	7	5	11	4246	5417	7809
DESpace2_LRT (individual test; 5 groups)	3	8	11	4991	5538	1322
DESpace2_LRT (individual test)	2	9	10	3546	3709	152
scran's `findMarkers`	6	4	9	4258	5420	7016
pseudobulk-based *Seurat*'s `FindMarkers`	6	4	9	5415	6434	2957
DESpace2_QLF (individual test; 5 groups)	3	6	9	4802	5557	372
scran's `findMarkers` (5 groups)	5	4	9	3800	4483	8732
spline-based *DESpace2_QLF* (5 groups)	5	4	7	1435	1558	130
spline-based *DESpace2_LRT* (5 groups)	4	4	6	1626	1742	261
spatialLIBD (5 groups)	2	1	3	5972	6630	6544

Supplementary Table 6: Summary of genes detected by each method in the *ARTISTA* dataset. The table shows the number of genes detected, among the top 200 results returned by each method. Gene sets were retrieved from The Human Protein Atlas website [Pontén et al., 2008] using the keywords “Healing” and “Regeneration”. These sets include 243 and 230 genes, respectively, of which 159 and 147 were present in our dataset. “Overall” represents the union of both sets (445 genes in total; 290 retained). The table also reports the average rank of genes in each list (lower values indicate higher significance), as well as the total number of genes called significant by each method.

Method	Identified in top 200	Average rank	# of GO terms
spline-based *DESpace2_ QLF* (5 groups)	6	75	196
DESpace2_ LRT (individual test; 5 groups)	6	27	2594
DESpace2_ QLF	4	64	244
spline-based *DESpace2_ LRT* (5 groups)	4	113	490
DESpace2_ LRT (individual test)	3	139	527
DESpace2_ QLF (5 groups)	2	33	1807
DESpace2_ LRT	2	102	1060
pseudobulk-based *Seurat*'s `FindMarkers`	2	110	4287
DESpace2_ QLF (individual test; 5 groups)	2	142	1091
spatialLIBD	2	173	577
DESpace2_ LRT (5 groups)	1	99	2557
Seurat's `FindMarkers`	0		6183
scran's `findMarkers`	0		5980
scran's `findMarkers` (5 groups)	0		6262
spatialLIBD (5 groups)	0		5289

Supplementary Table 7: Summary of Gene Ontology terms recovered by over-representation analysis in the *ARTISTA* dataset. For each method, significant genes were defined using adjusted p-value ≤ 0.01 and analyzed with `compareCluster` from the *clusterProfiler* R package [Yu et al., 2012]. The table reports, among the top 200 returned terms, the number of terms matching the keywords "wound healing" or "regeneration", their average rank, and the total number of terms returned by each method. Lower average ranks indicate higher-ranked enrichment results. The *DESpace2_ QLF* individual test was excluded because no genes passed adjusted p-value ≤ 0.01. For spline-based *DESpace2_ QLF* (5 groups), only 197 terms were returned, so keyword matching was evaluated over all 197 terms.

3 Supplementary Figures

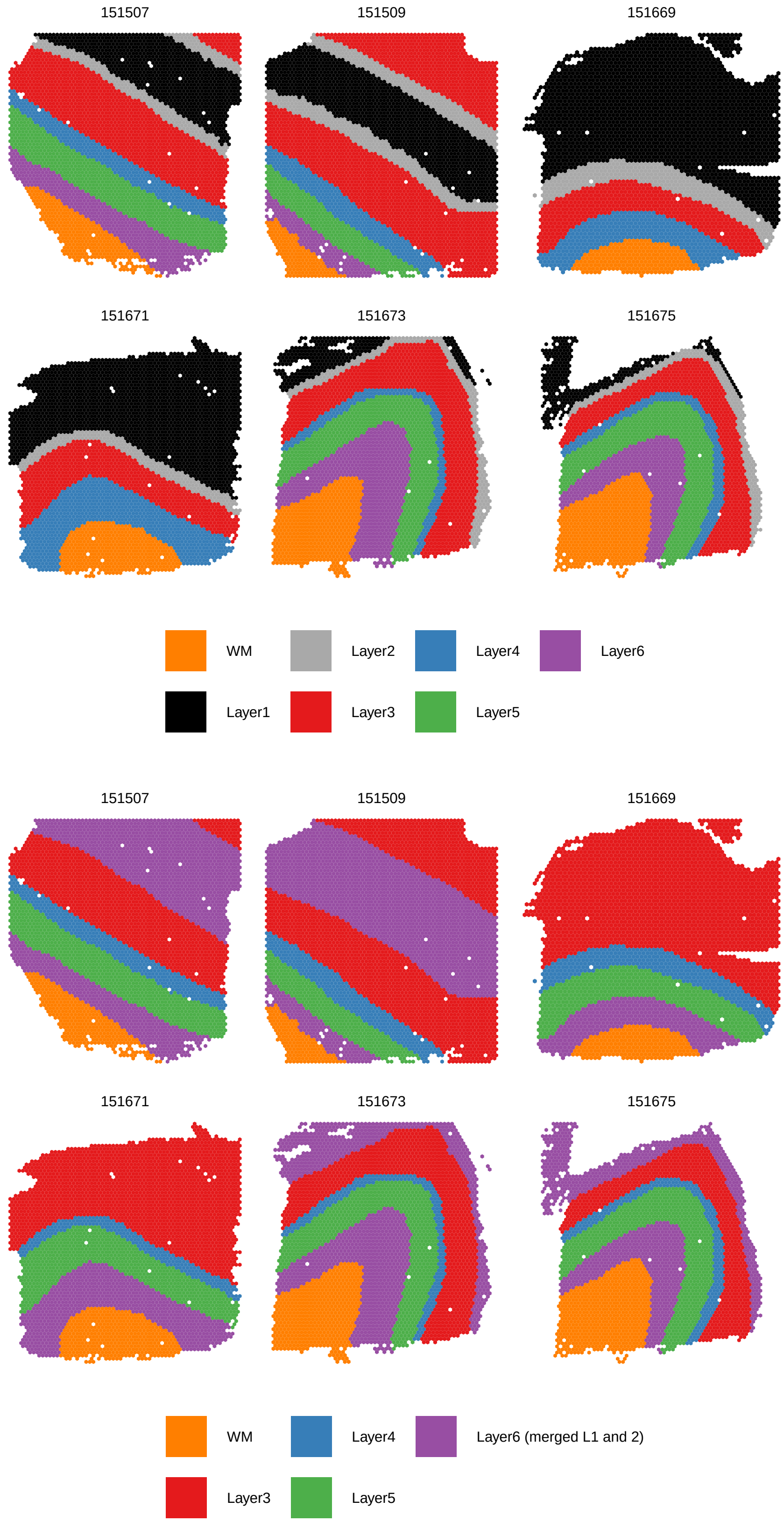

Supplementary Figure 1: Spatial domains for the *LIBD* dataset. Top: original pathologist annotations. Bottom: processed domains used in simulation, with Layers 1 and 2 merged into Layer 6 to harmonize domains across samples.

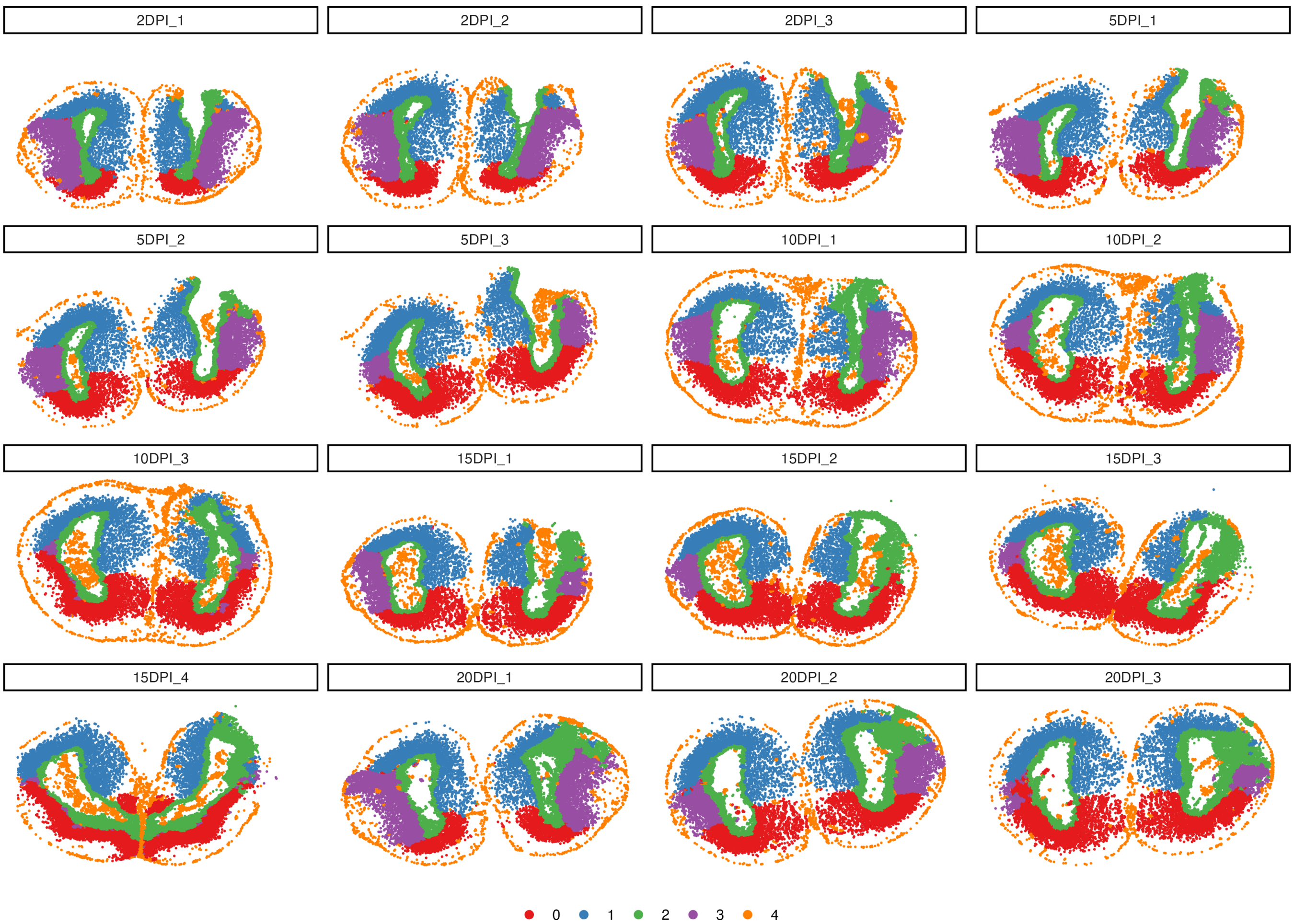

Supplementary Figure 2: Spatial domains for the *ARTISTA* dataset, obtained using joint clustering via *Banksy*. These domains were used for both real data analysis and simulation.

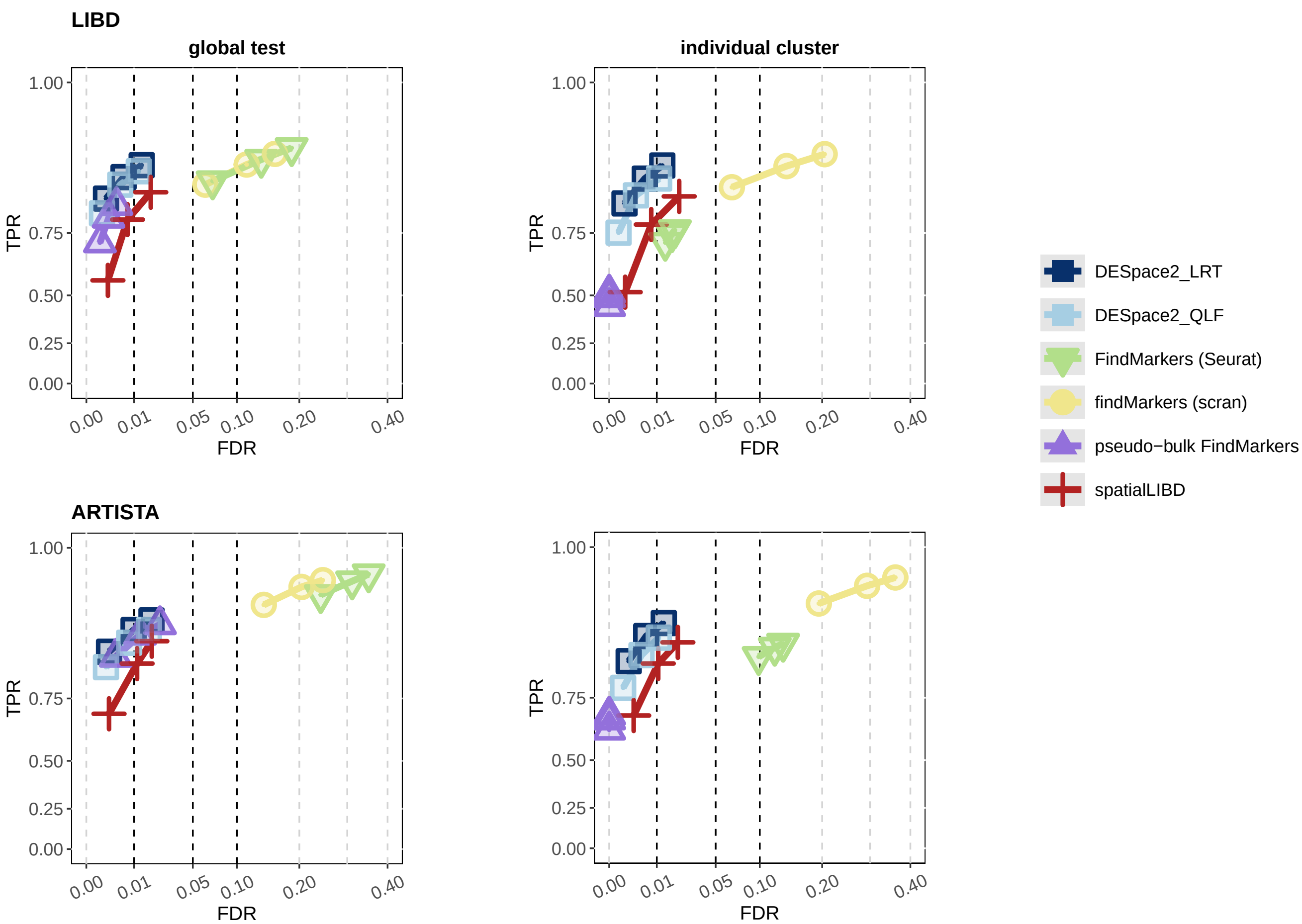

Supplementary Figure 3: TPR *vs.* FDR for DSP gene detection in simulations, where the main spatial cluster has lower abundance than the rest of the tissue. Top and bottom rows refer to the *LIBD* and *ARTISTA* data, respectively, while left and right columns indicate the global and individual-domain tests, respectively. Each method is evaluated at FDR thresholds of 0.01, 0.05, and 0.1; each point corresponds to one threshold.

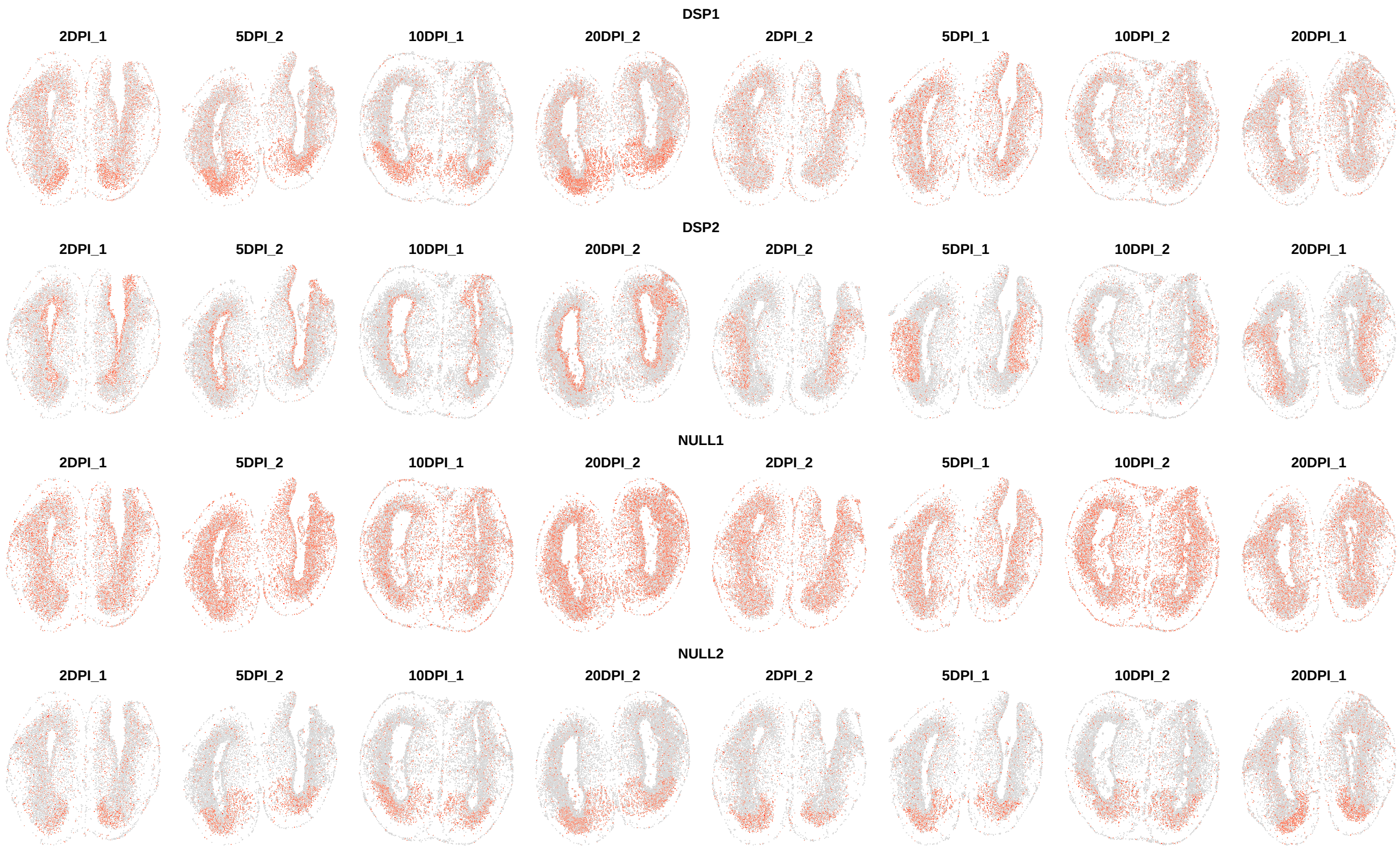

Supplementary Figure 4: Examples of simulated DSP genes from the *ARTISTA* dataset, categorized as *DSP1*, *DSP2*, *NULL1*, and *NULL2* (top to bottom), with the selected spatial domain showing higher abundance. The first four columns represent samples from condition 1, and the last four represent condition 2. Cells are colored by expression levels, from light grey (low) to red (high).

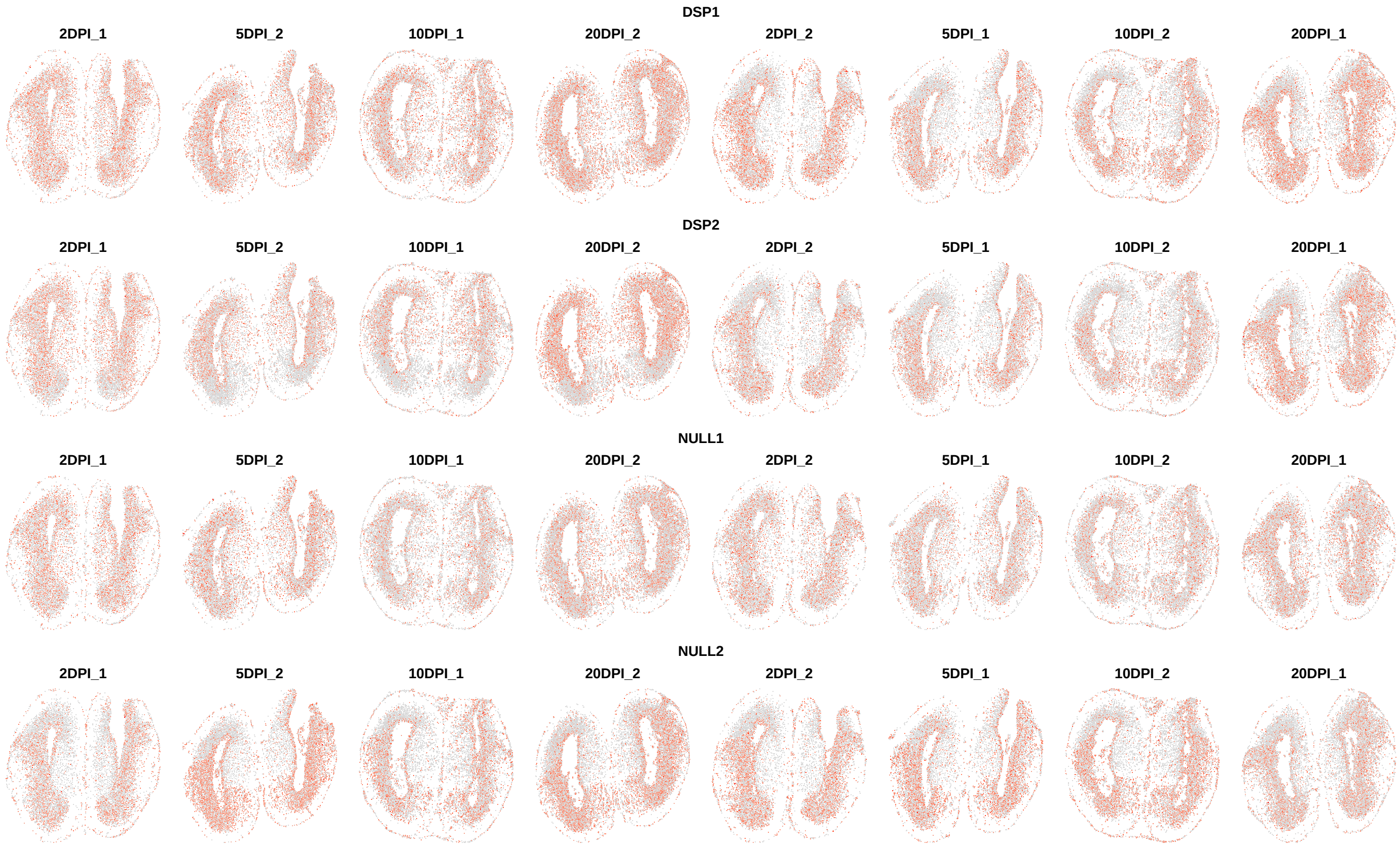

Supplementary Figure 5: Examples of simulated DSP genes from the *ARTISTA* dataset, categorized as *DSP1*, *DSP2*, *NULL1*, and *NULL2* (top to bottom), with the selected spatial domain showing lower abundance. The first four columns represent samples from condition 1, and the last four represent condition 2. Cells are colored by expression levels, from light grey (low) to red (high).

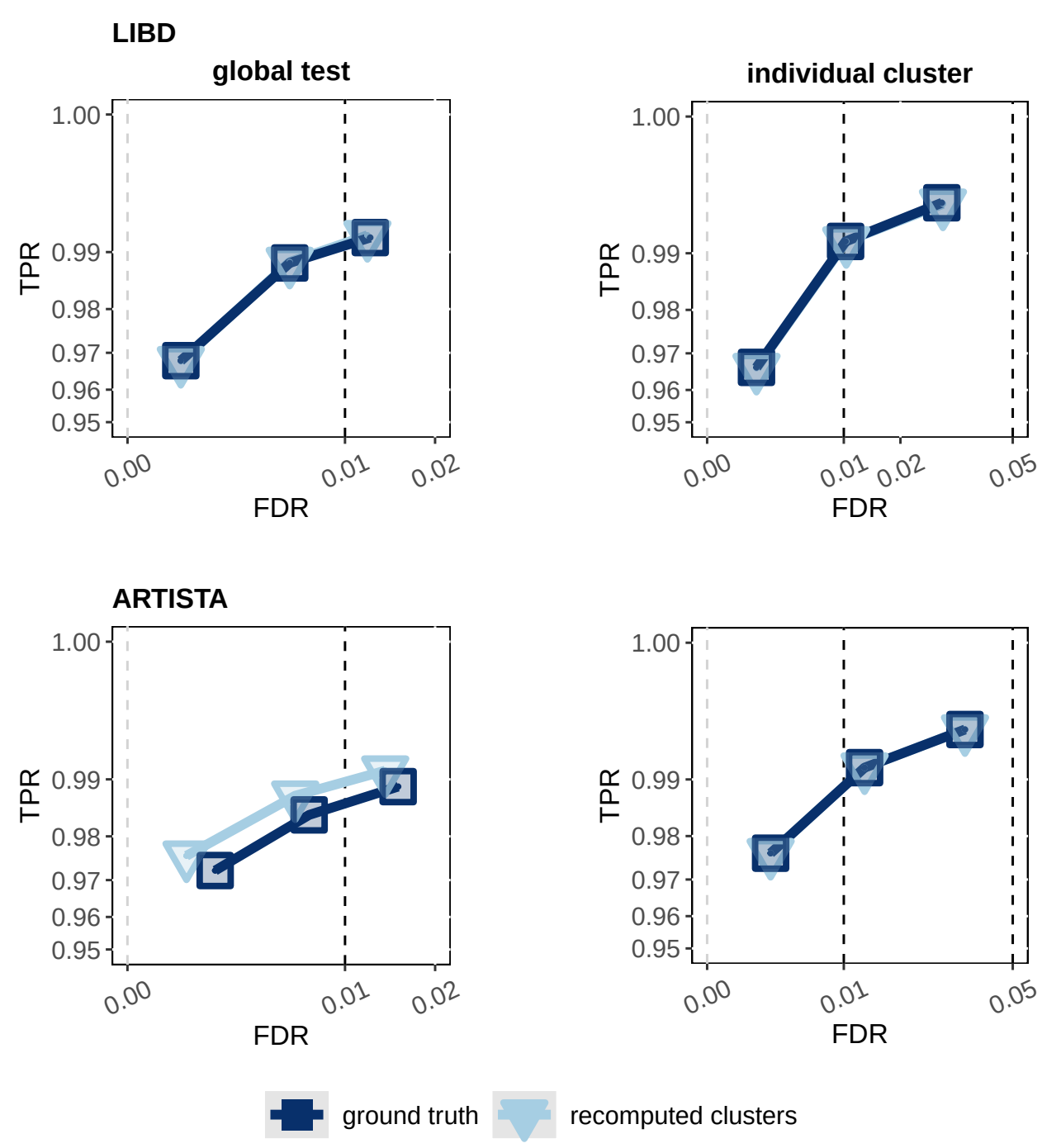

Supplementary Figure 6: TPR *vs.* FDR for DSP gene detection in our simulations. Top and bottom rows refer to the *LIBD* and *ARTISTA* data, respectively, while left and right columns indicate the global and individual-domain tests, respectively. Legend indicates the source of spatial clusters used by *DESpace2* with QLF tests: either *ground truth* (annotations used for simulation) or *recomputed clusters* (after simulation via *BayesSpace* for *LIBD* and *Banksy* for *ARTISTA*). Each method is evaluated at FDR thresholds of 0.01, 0.05, and 0.1; each point corresponds to one threshold.

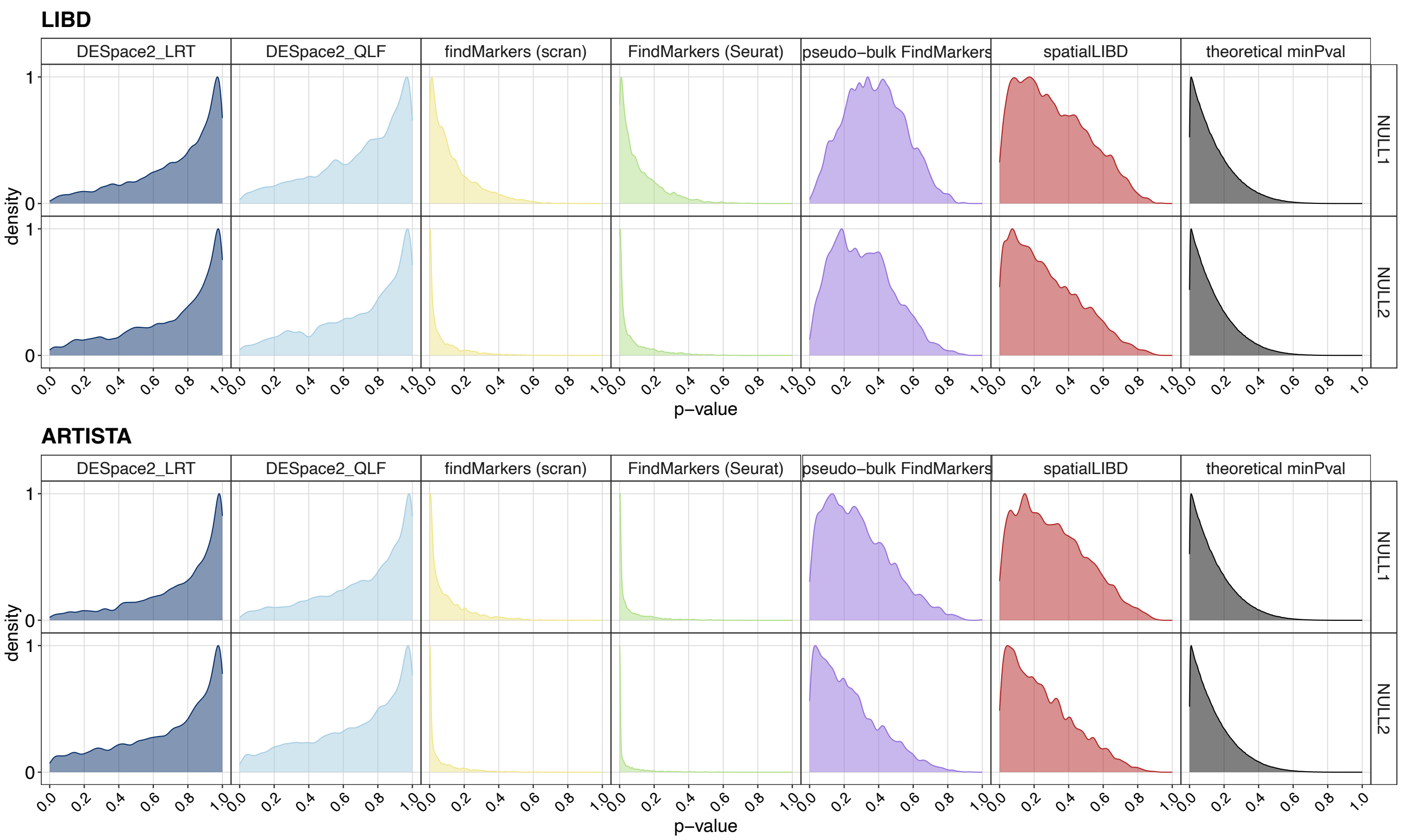

Supplementary Figure 7: Density of raw p-values in the null main simulations (i.e., no differences between conditions), based on *LIBD* and *ARTISTA* anchor dataset.

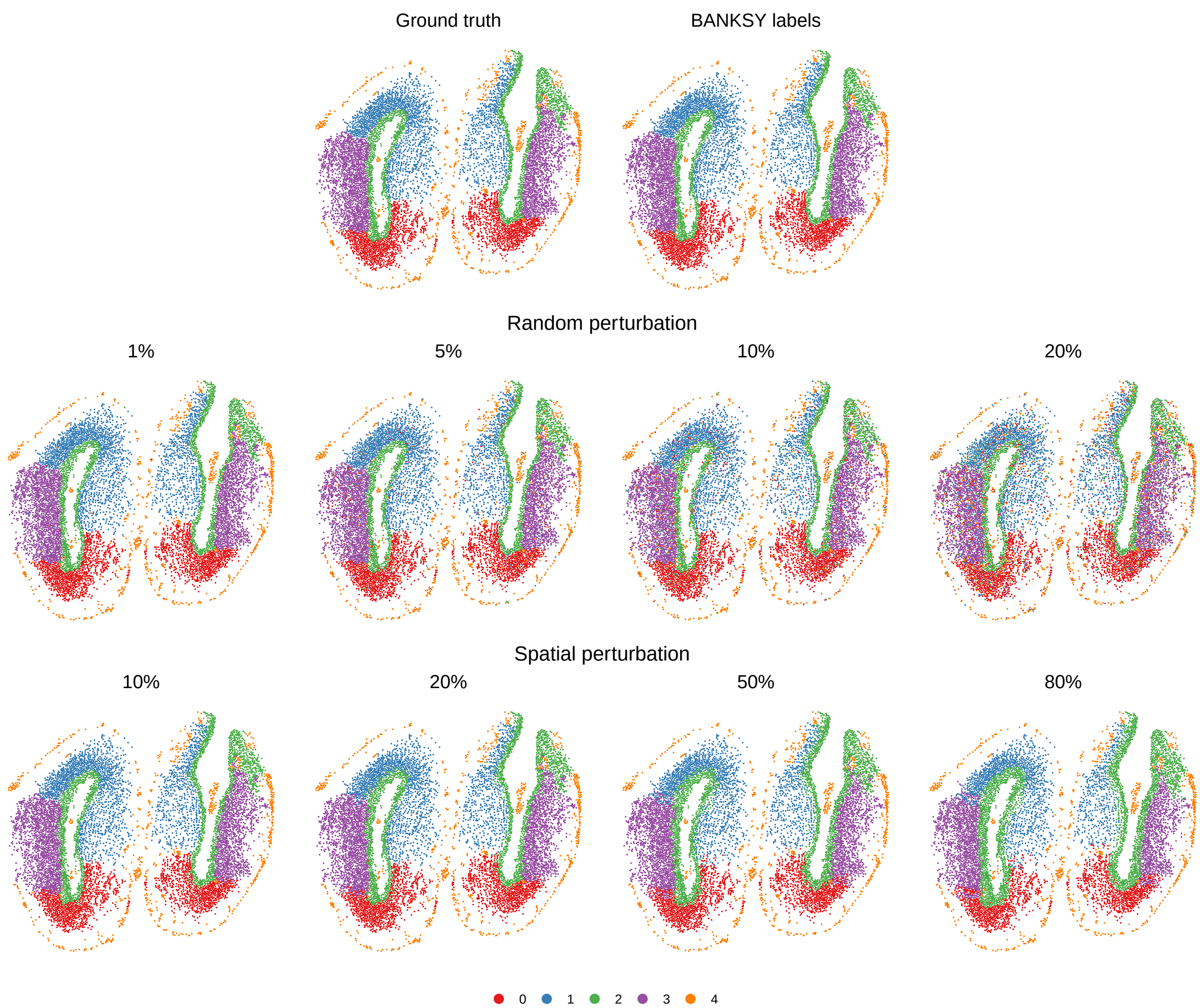

Supplementary Figure 8: Illustration of simulated mis-clustering scenarios in one representative sample from the *ARTISTA* dataset. The first row shows the ground-truth domain labels used to generate simulations and the original BANKSY clustering labels as references. Random perturbation scenarios were generated by reassigning 1%, 5%, 10%, and 20% of all cells to incorrect clusters. Spatial perturbation scenarios were generated by reassigning 10%, 20%, 50%, and 80% of boundary cells to incorrect neighboring clusters.

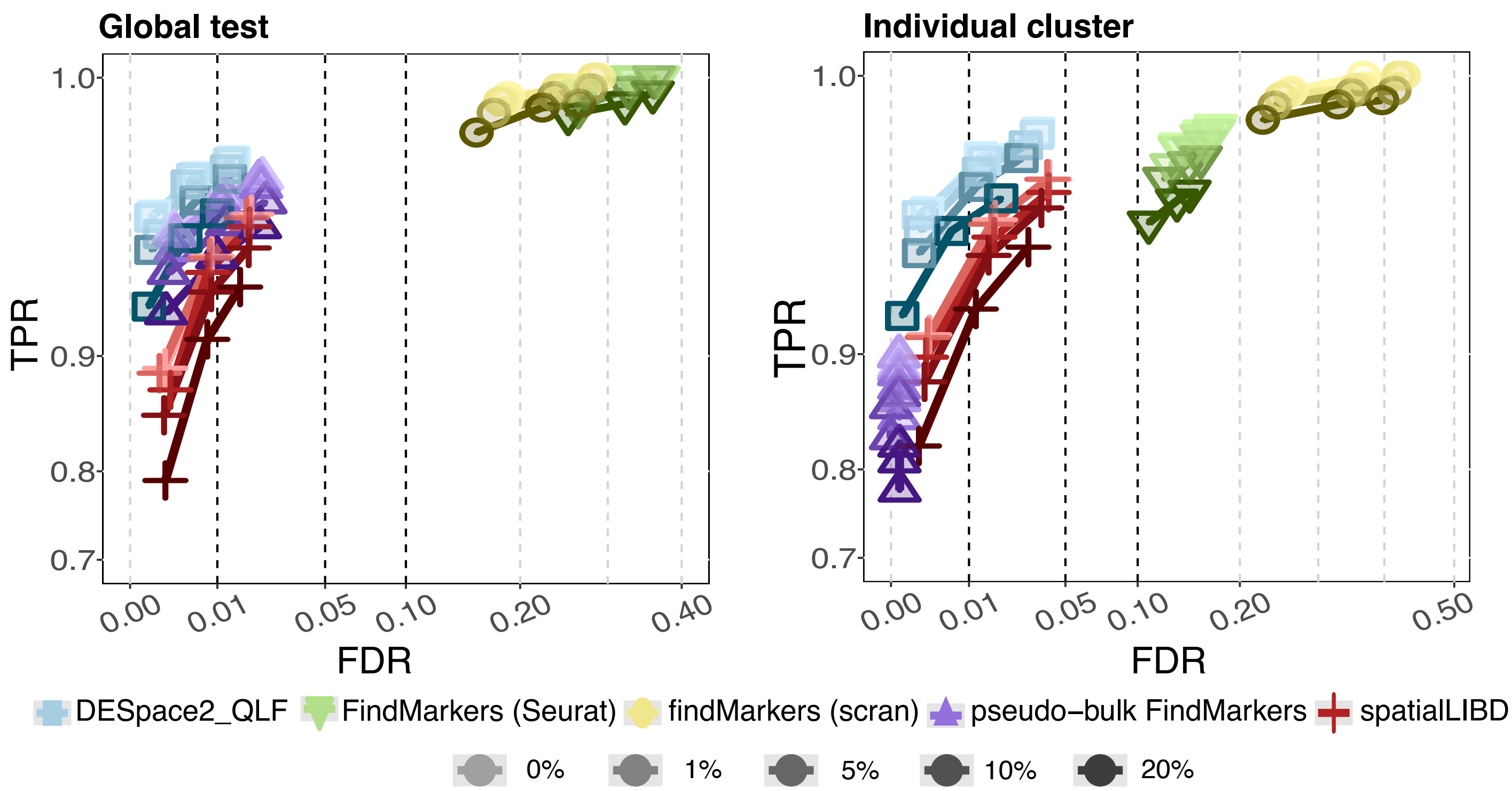

Supplementary Figure 9: TPR *vs.* FDR for DSP gene detection in sensitivity analyses using the *ARTISTA* dataset. A subset of cells (0%, 1%, 5%, 10%, and 20%) was randomly reassigned to incorrect clusters to simulate varying levels of mis-clustering. Right and left panels refer to the global and individual-domain tests, respectively. Each method was evaluated at nominal FDR thresholds of 0.01, 0.05, and 0.1; each point shows the observed TPR and FDR at one threshold.

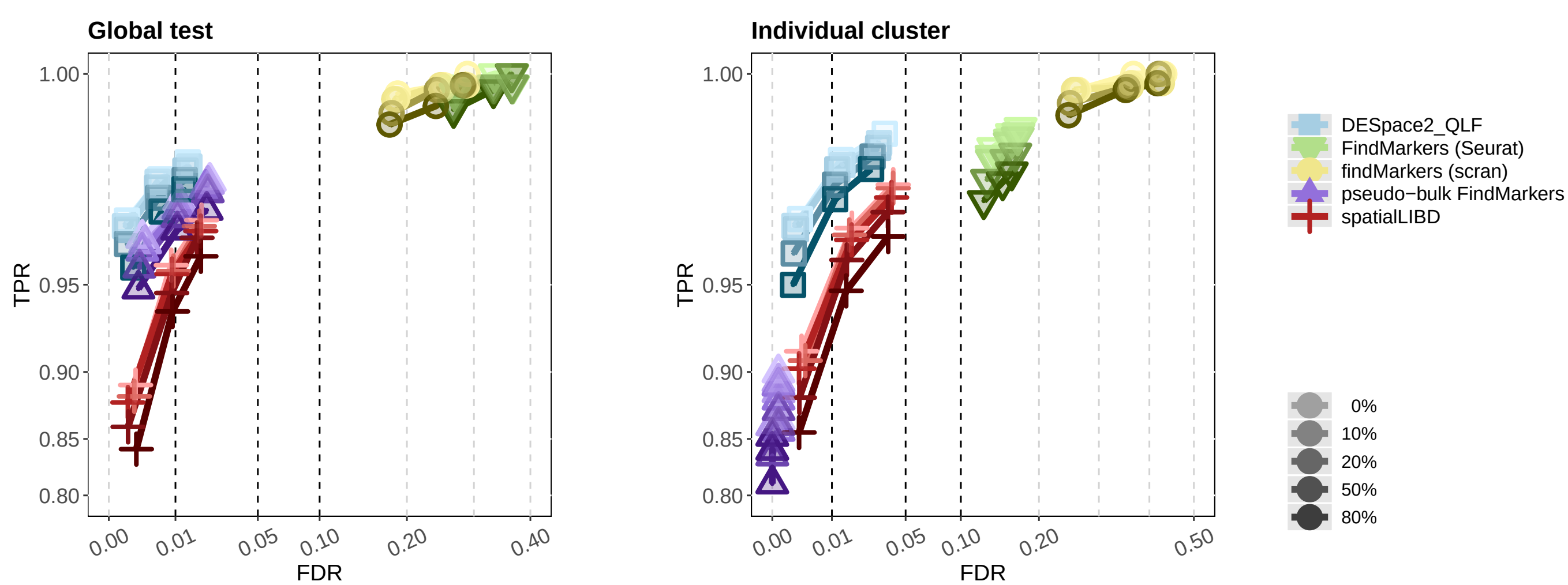

Supplementary Figure 10: TPR *vs.* FDR for DSP gene detection in sensitivity analyses using the *ARTISTA* dataset. A subset of boundary cells (0%, 10%, 20%, 50%, and 80%) was reassigned to incorrect neighboring clusters to simulate varying levels of mis-clustering. Right and left panels refer to the global and individual-domain tests, respectively. Each method was evaluated at nominal FDR thresholds of 0.01, 0.05, and 0.1; each point shows the observed TPR and FDR at one threshold.

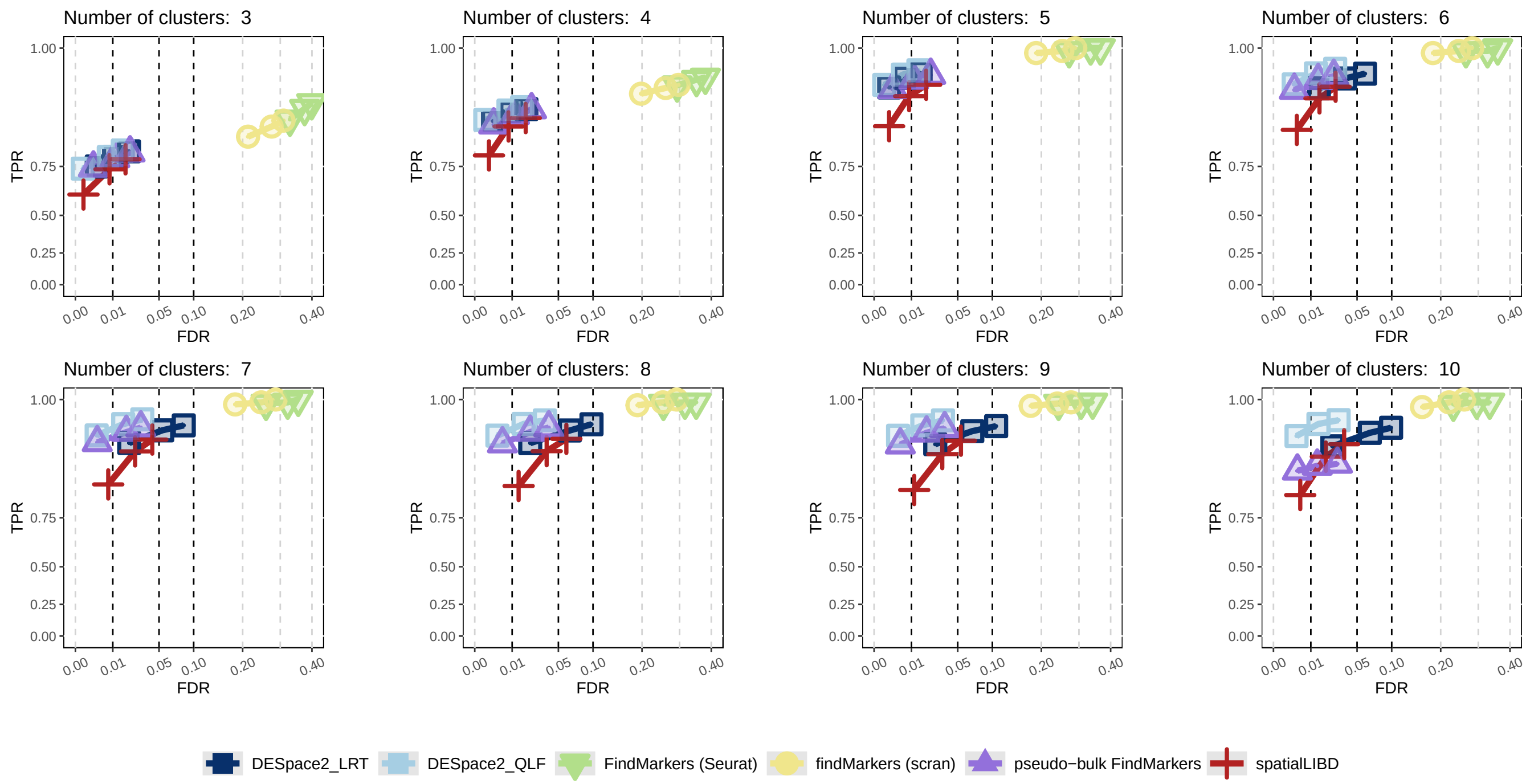

Supplementary Figure 11: TPR *vs.* FDR for DSP gene detection in sensitivity analyses using the *ARTISTA* dataset with the global test. Simulated dataset were generated with five clusters, but an incorrect number of clusters (ranging from three to ten) was specified when using *Banksy* clustering. Each method is evaluated at FDR thresholds of 0.01, 0.05, and 0.1; each point corresponds to one threshold.

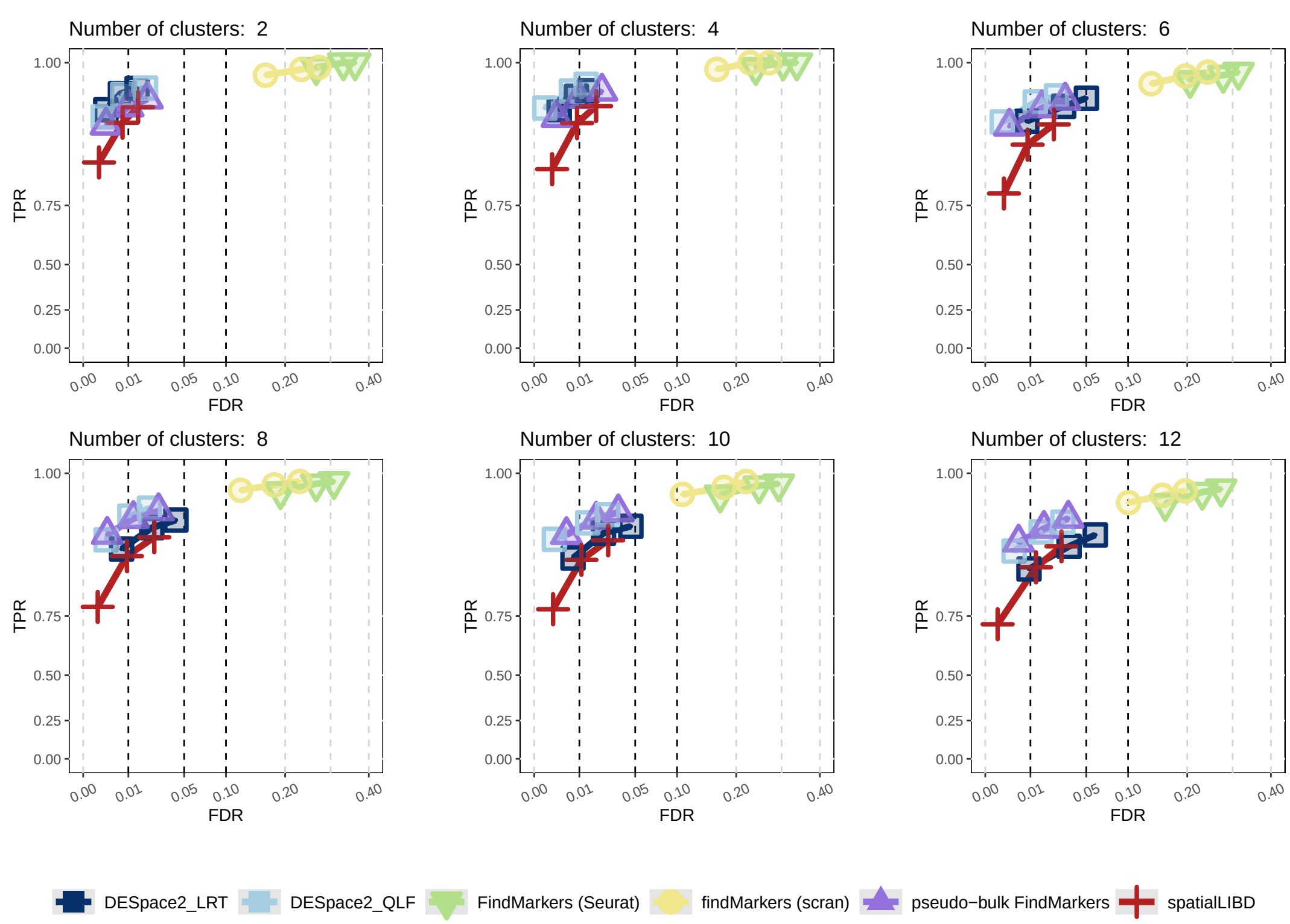

Supplementary Figure 12: TPR *vs.* FDR for DSP gene detection in sensitivity analyses using the *ARTISTA* dataset with the global test. Simulated datasets with varying cluster numbers were used to assess robustness to cluster resolution. Each method is evaluated at FDR thresholds of 0.01, 0.05, and 0.1; each point corresponds to one threshold.

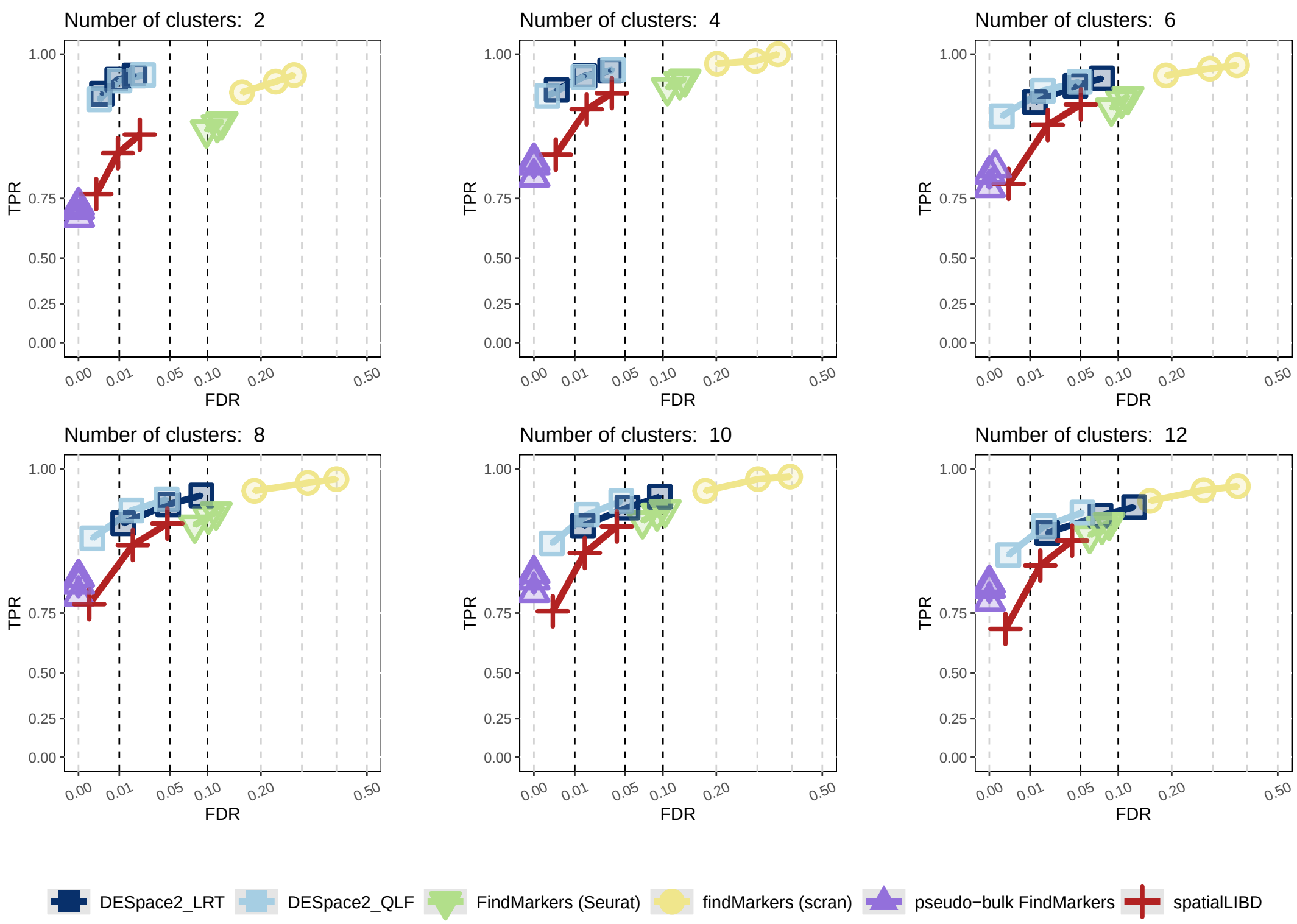

Supplementary Figure 13: TPR *vs.* FDR for DSP gene detection in sensitivity analyses using the *ARTISTA* dataset with the individual-domain test. Simulated datasets with varying cluster numbers were used to assess robustness to cluster resolution. Each method is evaluated at FDR thresholds of 0.01, 0.05, and 0.1; each point corresponds to one threshold.

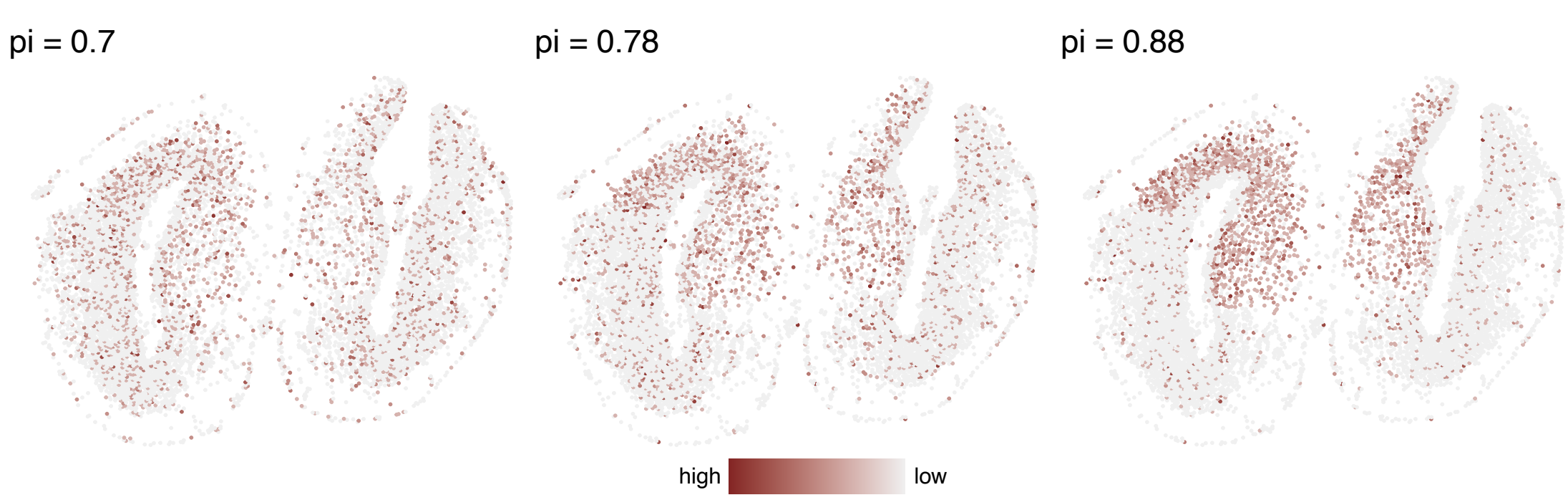

Supplementary Figure 14: Representative examples of the same simulated SVGs in one *ARTISTA* sample across increasing spatial signal strengths. The realized π values, sampled from Beta distributions with increasing means, are shown above each panel. Higher π produces stronger enrichment in the selected spatial domain. Cells are colored by expression levels, from light grey (low) to red (high).

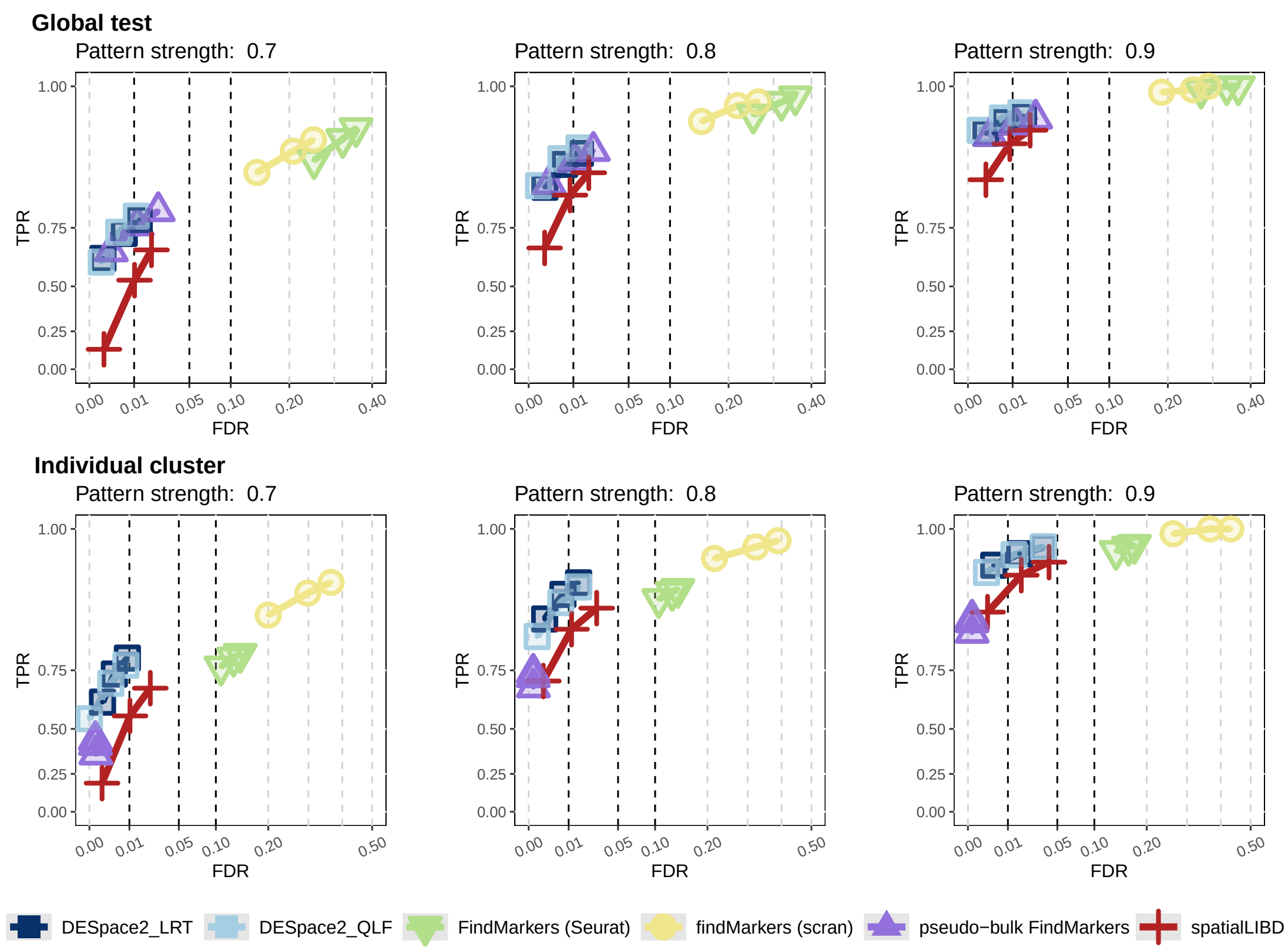

Supplementary Figure 15: TPR *vs.* FDR for DSP gene detection in sensitivity analyses using the *ARTISTA* dataset. Simulated datasets vary in spatial signal strength. Each method is evaluated at FDR thresholds of 0.01, 0.05, and 0.1; each point corresponds to one threshold.

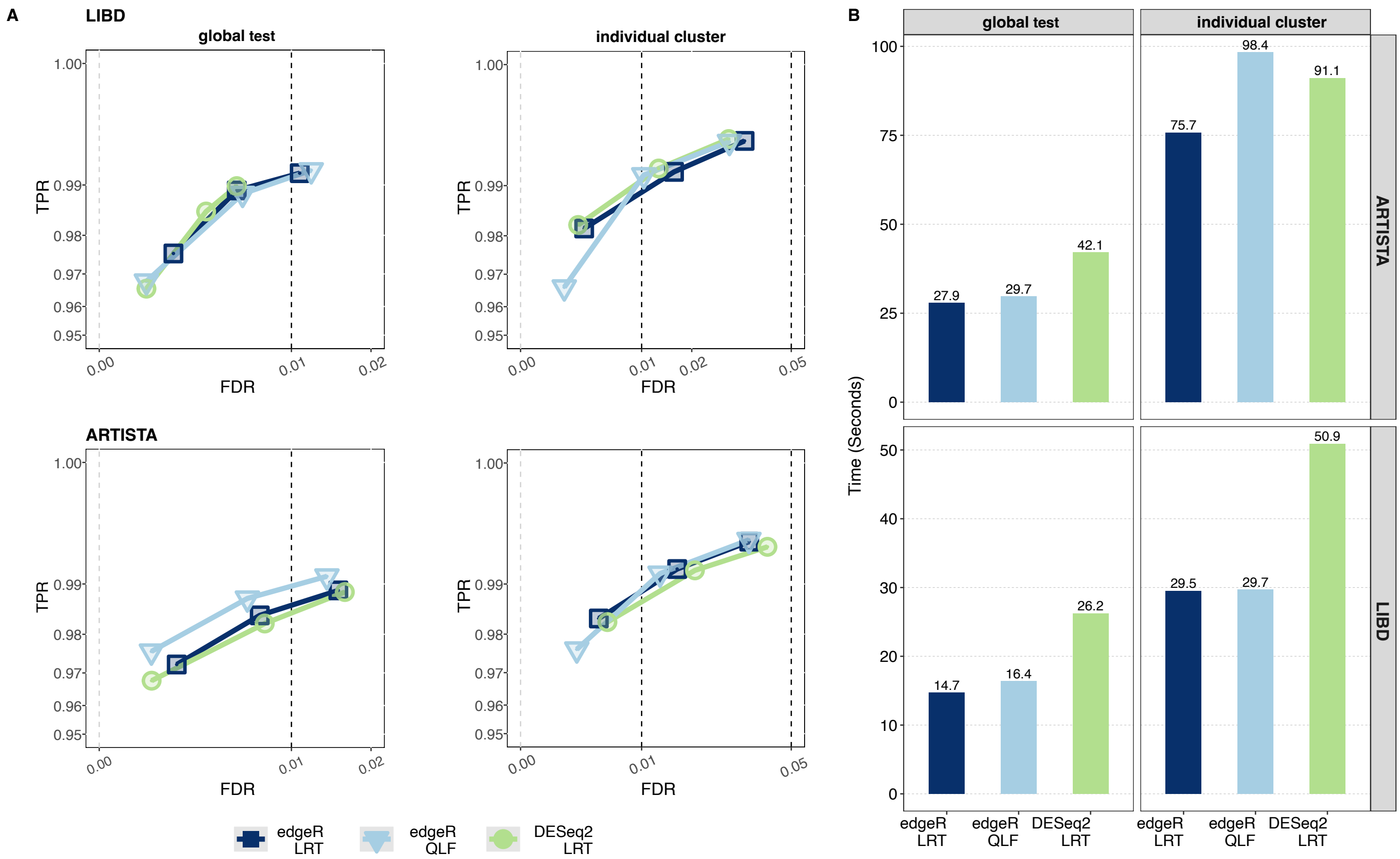

Supplementary Figure 16: A. TPR *vs.* FDR for DSP gene detection in the simulation study, comparing *DESpace2* implementations based on different differential gene expression (DGE) methods. Top and bottom rows correspond to the *LIBD* and *ARTISTA* datasets, respectively; left and right columns show the global and individual-domain tests, respectively. Each method is evaluated at FDR thresholds of 0.01, 0.05, and 0.1; each point corresponds to one threshold. B. Runtime in seconds for each *DESpace2* implementation.

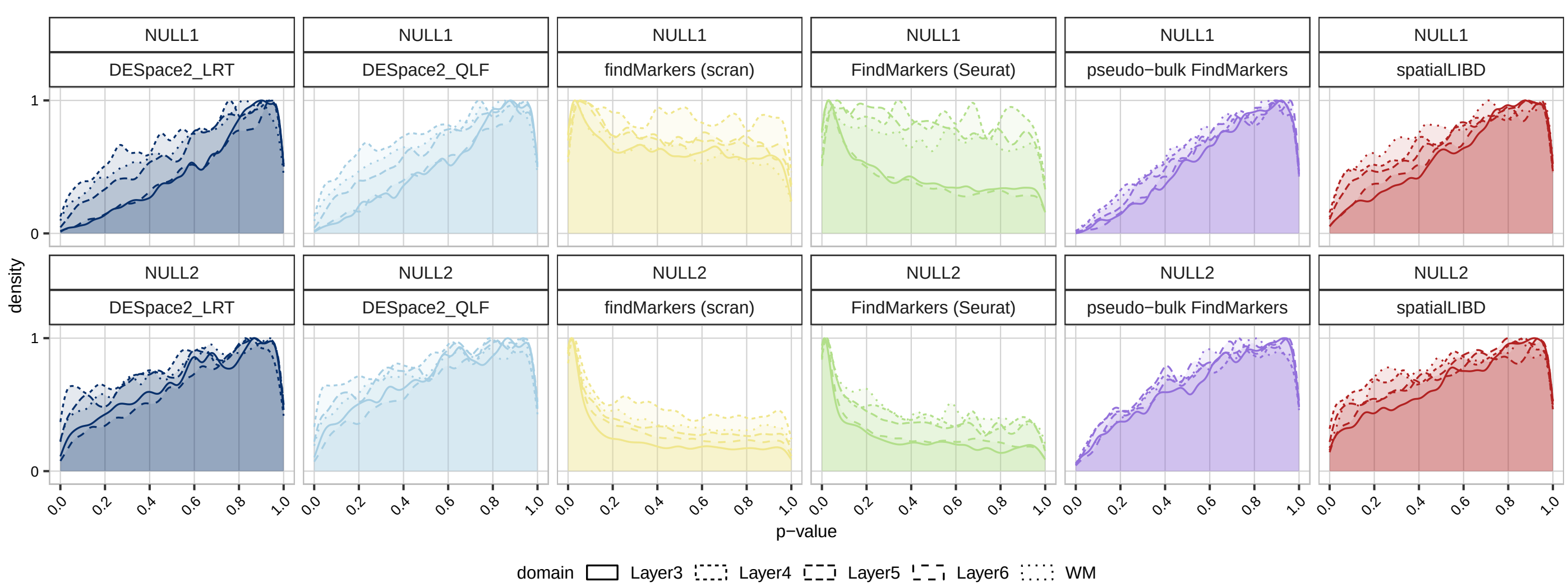

Supplementary Figure 17: Density of raw p-values in the null individual cluster simulations (i.e., no differences between groups), based on *LIBD* anchor dataset.

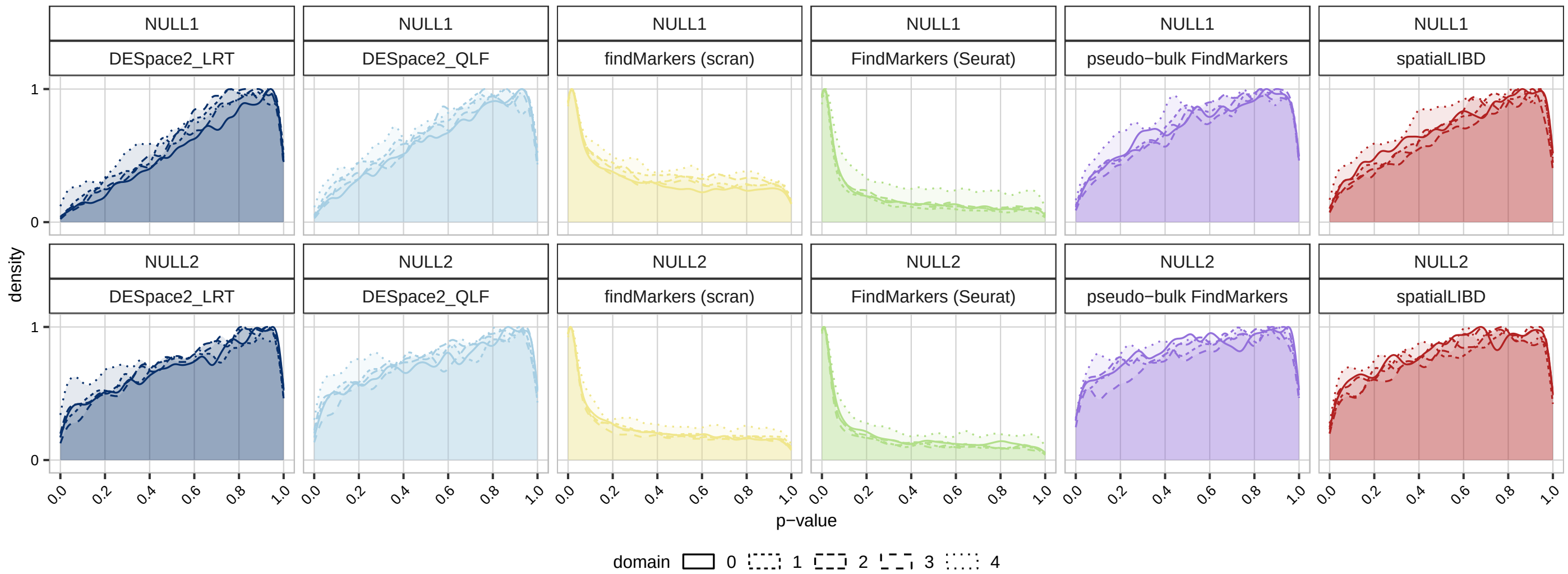

Supplementary Figure 18: Density of raw p-values in the null individual cluster simulations (i.e., no differences between groups), based on *ARTISTA* anchor dataset.

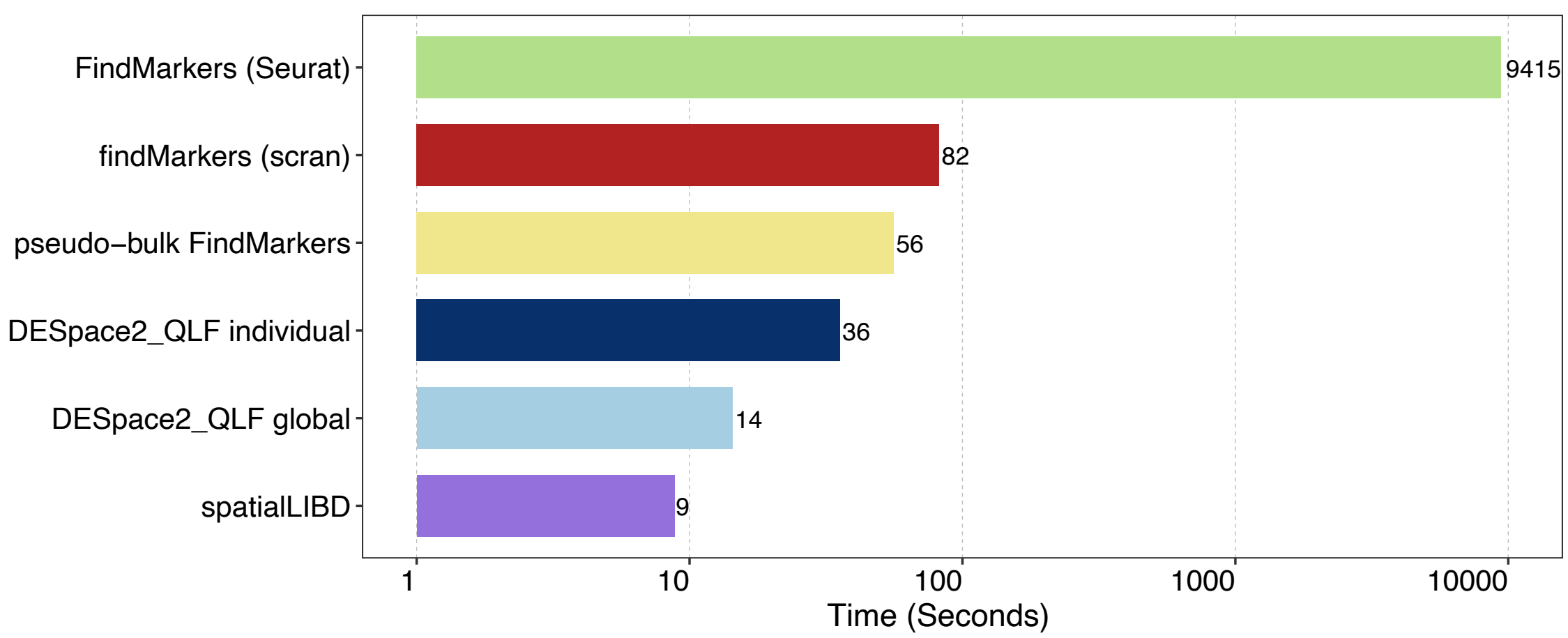

Supplementary Figure 19: Runtime (in seconds) for each method on the *ARTISTA* dataset using a 2-condition comparison (2 and 20 days post-injury) across six samples.

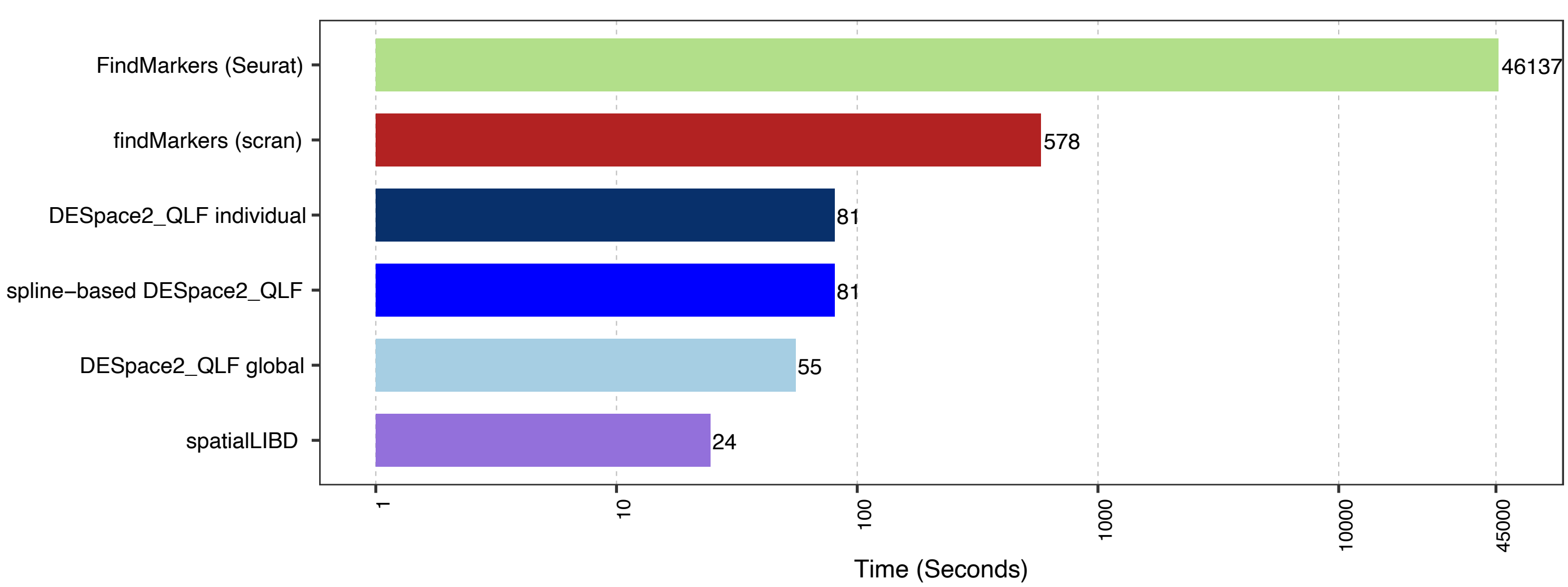

Supplementary Figure 20: Runtime (in seconds) for each method on the *ARTISTA* dataset using a 5-condition comparison (2, 5, 10, 15 and 20 days post-injury) across 16 samples.

References

Yoav Benjamini and Yosef Hochberg. Controlling the false discovery rate: a practical and powerful approach to multiple testing. *Journal of the Royal statistical society: series B (Methodological)*, 57(1):289–300, 1995.

Brenda Pardo, Abby Spangler, Lukas M Weber, Stephanie C Page, Stephanie C Hicks, Andrew E Jaffe, Keri Martinowich, Kristen R Maynard, and Leonardo Collado-Torres. spatialLIBD: an R/Bioconductor package to visualize spatially-resolved transcriptomics data. *BMC genomics*, 23(1).

Aaron TL Lun, Davis J McCarthy, and John C Marioni. A step-by-step workflow for low-level analysis of single-cell RNA-seq data with Bioconductor. *F1000Research*, 5:2122, 2016.

Rahul Satija, Jeffrey A Farrell, David Gennert, Alexander F Schier, and Aviv Regev. Spatial reconstruction of single-cell gene expression data. *Nature biotechnology*, 33(5):495–502, 2015.

Simon Anders, Alejandro Reyes, and Wolfgang Huber. Detecting differential usage of exons from rna-seq data. *Nature Precedings*, pages 1–1, 2012.

Michael I Love, Wolfgang Huber, and Simon Anders. Moderated estimation of fold change and dispersion for RNA-seq data with DESeq2. *Genome biology*, 15: 1–21, 2014.

Robert A Amezquita, Aaron TL Lun, Etienne Becht, Vince J Carey, Lindsay N Carpp, Ludwig Geistlinger, Federico Marini, Kevin Rue-Albrecht, Davide Risso, Charlotte Soneson, et al. Orchestrating single-cell analysis with bioconductor. *Nature methods*, 17(2):137–145, 2020.

Davis J McCarthy, Kieran R Campbell, Aaron TL Lun, and Quin F Wills. Scater: pre-processing, quality control, normalization and visualization of single-cell RNA-seq data in R. *Bioinformatics*, 33(8):1179–1186, 2017.

Ruben Dries, Qian Zhu, Rui Dong, Chee-Huat Linus Eng, Huipeng Li, Kan Liu, Yuntian Fu, Tianxiao Zhao, Arpan Sarkar, Feng Bao, et al. Giotto: a toolbox for integrative analysis and visualization of spatial expression data. *Genome biology*, 22:1–31, 2021.

Kristen R Maynard, Leonardo Collado-Torres, Lukas M Weber, Cedric Uytingco, Brianna K Barry, Stephen R Williams, Joseph L Catallini, Matthew N Tran, Zachary Besich, Madhavi Tippani, et al. Transcriptome-scale spatial gene expression in the human dorsolateral prefrontal cortex. *Nature neuroscience*, 24(3): 425–436, 2021.

Mark D Robinson, Davis J McCarthy, and Gordon K Smyth. edgeR: a Bioconductor package for differential expression analysis of digital gene expression data. *bioinformatics*, 26(1):139–140, 2010.

Arthur Liberzon, Aravind Subramanian, Reid Pinchback, Helga Thorvaldsdóttir, Pablo Tamayo, and Jill P Mesirov. Molecular signatures database (msigdb) 3.0. *Bioinformatics*, 27(12):1739–1740, 2011.

Arthur Liberzon, Chet Birger, Helga Thorvaldsdóttir, Mahmoud Ghandi, Jill P Mesirov, and Pablo Tamayo. The molecular signatures database hallmark gene set collection. *Cell systems*, 1(6):417–425, 2015.

Fredrik Pontén, Karin Jirström, and Matthias Uhlen. The Human Protein Atlas—a tool for pathology. *The Journal of Pathology: A Journal of the Pathological Society of Great Britain and Ireland*, 216(4):387–393, 2008.

Guangchuang Yu, Li-Gen Wang, Yanyan Han, and Qing-Yu He. clusterprofiler: an r package for comparing biological themes among gene clusters. *Omics: a journal of integrative biology*, 16(5):284–287, 2012.
